# Activity of Lymphostatin, A Lymphocyte Inhibitory Virulence Factor of Pathogenic *Escherichia coli*, is Dependent on a Cysteine Protease Motif

**DOI:** 10.1016/j.jmb.2021.167200

**Published:** 2021-09-17

**Authors:** Andrew G. Bease, Elizabeth A. Blackburn, Cosmin Chintoan-Uta, Shaun Webb, Robin L. Cassady-Cain, Mark P. Stevens

**Affiliations:** a The Roslin Institute and Royal (Dick) School of Veterinary Studies, University of Edinburgh, Midlothian EH25 9RG, United Kingdom; b Edinburgh Protein Production Facility, University of Edinburgh, Michael Swann Building, King’s Buildings, Edinburgh EH9 3BF, United Kingdom; c Bioinformatics Core Facility, Institute of Cell Biology, University of Edinburgh, Michael Swann Building, King’s Buildings, Edinburgh, EH9 3BF, United Kingdom

**Keywords:** LifA, protein toxin, endosome acidification, endopeptidase, protein processing, CD, circular dichroism, ConA, concanavalin A, CPD, cysteine protease domain, Efa1, EHEC factor for adherence 1, EHEC, enterohaemorrhagic *E. coli*, EPEC, enteropathogenic *E. coli*, IMAC, ion metal affinity chromatography, LCT, large clostridial toxin, LifA, lymphocyte inhibitory factor A, SAXS, small angle X-ray scattering, SEC, size-exclusion chromatography

## Abstract

Lymphostatin (LifA) is a 366 kDa protein expressed by attaching & effacing *Escherichia coli*. It plays an important role in intestinal colonisation and inhibits the mitogen- and antigen-stimulated proliferation of lymphocytes and the synthesis of proinflammatory cytokines. LifA exhibits N-terminal homology with the glycosyltransferase domain of large clostridial toxins (LCTs). A DTD motif within this region is required for lymphostatin activity and binding of the sugar donor uridine diphosphate *N*-acetylglucosamine. As with LCTs, LifA also contains a cysteine protease motif (C1480, H1581, D1596) that is widely conserved within the YopT-like superfamily of cysteine proteases. By analogy with LCTs, we hypothesised that the CHD motif may be required for intracellular processing of the protein to release the catalytic N-terminal domain after uptake and low pH-stimulated membrane insertion of LifA within endosomes. Here, we created and validated a C1480A substitution mutant in LifA from enteropathogenic *E. coli* strain E2348/69. The purified protein was structurally near-identical to the wild-type protein. In bovine T lymphocytes treated with wild-type LifA, a putative cleavage product of approximately 140 kDa was detected. Appearance of the putative cleavage product was inhibited in a concentration-dependent manner by bafilomycin A1 and chloroquine, which inhibit endosome acidification. The cleavage product was not observed in cells treated with the C1480A mutant of LifA. Lymphocyte inhibitory activity of the purified C1480A protein was significantly impaired. The data indicate that an intact cysteine protease motif is required for cleavage of lymphostatin and its activity against T cells.

## Highlights

• LifA shares a cysteine protease motif with bacterial toxins and secreted effectors.
• C1480A substituted LifA has reduced inhibitory activity against T cells.
• LifA is cleaved in T cells and this requires C1480 and endosome acidification.

## Graphical abstract

**Figure:**
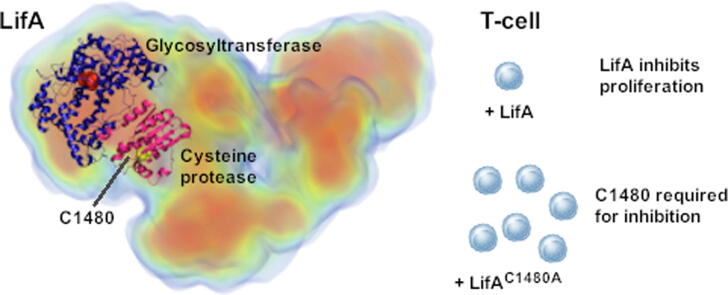

## Introduction

Gastrointestinal bacterial infections have a substantial impact on both human and animal health. Two prominent pathogens are enteropathogenic *Escherichia coli* (EPEC) and enterohaemorrhagic *E. coli* (EHEC), which are estimated to cause over 14 million human infections per annum.^1^ EHEC differ from EPEC by the production of one or more Shiga toxins that cause damage to vascular endothelia that can lead to haemolytic uraemic syndrome. EPEC and EHEC require a Type III secretion system (T3SS) for intestinal colonisation that is encoded by the locus of enterocyte effacement (LEE).^2^ The T3SS injects a set of effector proteins into enterocytes that subvert cellular processes to the benefit of the pathogen, resulting in intimate adherence of the bacteria to the apical surface of host cells on raised actin-rich pedestals and effacement of microvilli.^3^ EPEC and EHEC express a number of additional virulence factors that support infection,^4^ one of which is the protein lymphostatin, which is encoded proximal to the LEE in some strains. It is known to be important for intestinal colonisation in cattle by EHEC serogroup O5, O26 and O111 strains^5^, ^6^ and for colonisation of mice by the murine attaching & effacing pathogen *Citrobacter rodentium*.^7^ Although the precise mode of action of lymphostatin remains ill-defined, it has been reported to act as an inhibitor of the activation of T lymphocytes from a number of species and tissues.^5^, ^8^, ^9^, ^10^, ^11^, ^12^ Our laboratory has found that lymphostatin is active against both CD4 and CD8 subsets, exhibits modest activity against B-cells but has very little effect on NK cells, and inhibits the mitogen-activated synthesis of a range of bovine proinflammatory cytokines.^11^ Lymphostatin has also been shown to influence adherence in some strains,^13^ possibly as a consequence of effects on Type III secretion.^5^, ^6^ Further, although LifA has been reported to be secreted via the LEE-encoded T3SS^14^ and is involved in pedestal formation in EPEC strains that lack multiple effectors,^13^ its activity against lymphocytes does not require injection and can be observed following treatment with highly purified protein.^12^

**Figure 1:**
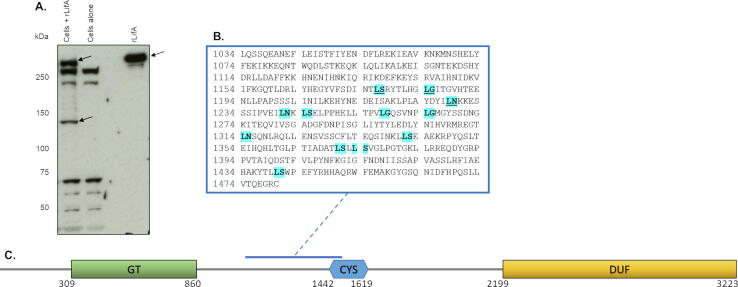
Lymphostatin is processed in bovine T lymphocytes. (a) Western blots reveal a putative cleavage product with a molecular weight of approximately 140 kDa in the lysates of bovine T lymphocytes treated with WT rLifA. An absolute number of 1.2 × 10^7^ cells were treated with/without WT rLifA for 1 h. A volume equivalent to 2 × 10^6^ cells was run in each well. Full-length WT rLifA (~366 kDa) and the putative cleavage product are indicated by arrows. (b) Predicted cleavage sites (in bold, highlighted cyan) of lymphostatin, based on large clostridial toxin cleavage sites, that lie between the first amino acid after the end of the predicted glycosyltransferase domain and the catalytic C1480. Underlined amino acids represent cleavage sites that would produce an N-terminal product of approximately the size of the observed putative cleavage product. (c) A schematic representation of the domain organisation of lymphostatin. GT: glycosyltransferase domain, CYS:cysteine protease domain, DUF:domain of unknown function. A blue line highlights the region of putative cleavage.

Lymphostatin is one of the largest *E. coli* proteins known at 366 kDa (Figure 1) and is a putative glycosyltransferase with homology over the N-terminal third to large clostridial toxins (LCTs).^10^, ^11^, ^12^, ^13^ The LCTs TcdA and TcdB are known to glucosylate Rho-family GTPases that regulate the actin cytoskeleton, ultimately inducing morphological changes and cytotoxicity.^16^ LCTs from *Clostridium novyi* (TcnA) and *C. perfringens* (TpeL) have been reported to transfer *N*-acetylglucosamine (GlcNAc) to Rho family and Ras GTPases from a uridine diphosphate (UDP) sugar donor.^17^, ^18^ Previously, we have shown that recombinant lymphostatin is able to bind uridine diphosphate *N*-acetylglucosamine (UDP-GlcNAc),^12^ and that a DTD motif in the N-terminal glycosyltransferase domain is essential for both binding of the sugar donor and inhibition of mitogen-activated T cell proliferation.^12^

In addition to possession of a glycosyltransferase domain, lymphostatin also shares a putative cysteine protease motif with LCTs.^13^, ^19^ This motif is characterised by the presence of a catalytic triad of Cys-His-Asp that is widely conserved in the C58 family of bacterial virulence factors that contain a YopT-like domain^19^, ^20^ (C1480, H1581, D1596 in the prototype LifA protein from EPEC strain E2348/69). In LCTs, the cysteine protease motif is required to facilitate intracellular autocatalytic cleavage of the protein to release the glycosyltransferase domain.^21^ Briefly, LCTs bind at the surface of the target cell and undergo receptor-mediated endocytosis. As the endosome matures, the internal pH of the endosome drops, leading to structural changes that promote insertion of the protein across the endosomal membrane. The cysteine protease motif mediates the autocatalytic cleavage of the protein, using inositol hexakisphosphate as a co-factor, allowing the release of the glycosyltransferase domain into the cytosol.^22^ Some bacterial virulence factors, such as YopT, use their cysteine protease motif to cleave cellular targets, thereby producing cytotoxic effects.^19^ However, published studies to date have not identified direct cytotoxicity associated with LifA treatment (e.g. release of lactate dehydrogenase or altered cellular morphology or viability),^10^, ^12^ suggesting that lymphostatin functions in a different manner.

LifA can be encoded within the same pathogenicity island as Type III secreted effectors with glycosyltransferase (NleB) and cysteine protease (EspL) activities in the prototype EPEC strain E2348/69.^23^ However, sequencing of non-O157 STEC strains has reported that the location of the gene and composition of the pathogenicity island encoding NleB and EspL can differ.^24^ EspL uses its cysteine protease activity to cleave RHIM-containing proteins (RIPK1, RIPK3, TRIF and ZBP1/DAI) to prevent lipopolysaccharide or polyI:C-induced inflammatory signalling.^25^ The presence of an additional cysteine protease motif in lymphostatin with potentially different specificity is intriguing.

The cysteine protease motif of lymphostatin has previously been reported to be required for colonic colonisation of mice by *C. rodentium* and the induction of hyperplasia.^7^ However, the method used to generate a cysteine protease motif mutant in this study resulted in the introduction of a stop codon, causing protein truncation rather than an in-frame deletion.^6^, ^26^ A second study by the same authors created an in-frame deletion of the *C. rodentium* LifA cysteine protease motif, but the effect on gut colonisation in mice and lymphostatin activity was not reported.^26^ Separately, it was reported that a chromosomal deletion of the cysteine protease motif in EPEC E2348/69 *lifA* impaired the ability of cell lysates to inhibit the concanavalin A (ConA)-simulated proliferation of lymphocytes.^6^ The deletion encompassed a 369 bp region that included residues C1480, H1581 and D1596 and the impact of a significant change of this nature on the structure of the protein was not studied.

With the exception of these studies, little has been done to investigate the role that the cysteine protease motif plays in the activity or processing of lymphostatin. We hypothesised that a cleavage event, similar to that observed with LCTs, is required for the full activity of lymphostatin on T cells. We investigated this by analysing the processing of LifA in T cells, structural modelling, site-directed mutagenesis of the predicted catalytic cysteine residue, purification and biophysical analysis of the altered protein, and analysis of the processing and activity of the altered protein in T cells.

## Results

### A processed form of lymphostatin appears in bovine T cells

In order to investigate the hypothesis that lymphostatin is processed in T cells, we analysed lysates of T cells that had been treated with recombinant LifA (rLifA) from EPEC E2348/69 by western blotting using a polyclonal antibody that is known to recognise multiple domains of the protein (see Footnotes). If the cysteine protease domain (CPD) of lymphostatin mediates autocatalytic cleavage in a manner similar to the LCTs, then we predicted that this cleavage should yield a fragment of at least 100 kDa encompassing the N-terminal glycosyltransferase domain that we previously localised to residues 309–860,^12^ and another fragment of at least 265 kDa encompassing the rest of the molecule. In T cells treated with wild-type rLifA (WT rLifA) we observed a single specific and reproducible species reactive to anti-LifA antibodies running at approximately 140 kDa in SDS-PAGE (Figure 1(A) and (C)), suggesting that lymphostatin is processed in T cells. The 140 kDa species and the expected C-terminal His-tagged species of 225 kDa were not detected with anti-6 × His antibodies, possibly owing to the limit of sensitivity of the assay, retention in endosomes and degradation or masking by a T cell protein reactive to anti-LifA antibodies. Full-length intact rLifA (366 kDa) and non-specific T cell species (at approximately 270, 230, 65 kDa and below) were also detected. LCTs autocatalytically cleave between conserved leucine residues and either serine, glycine or asparagine residues located between the glycosyltransferase and cysteine protease motifs.^27^, ^28^, ^29^ Thirteen such potential cleavage sites exist at the following residues in strain E2348/69 lymphostatin: 1087, 1176, 1184, 1228, 1241, 1244, 1257, 1264, 1314, 1341, 1370, 1373 and 1440 (Figure 1(B)).

### Modelling the putative cysteine protease domain of lymphostatin

**Figure 2:**
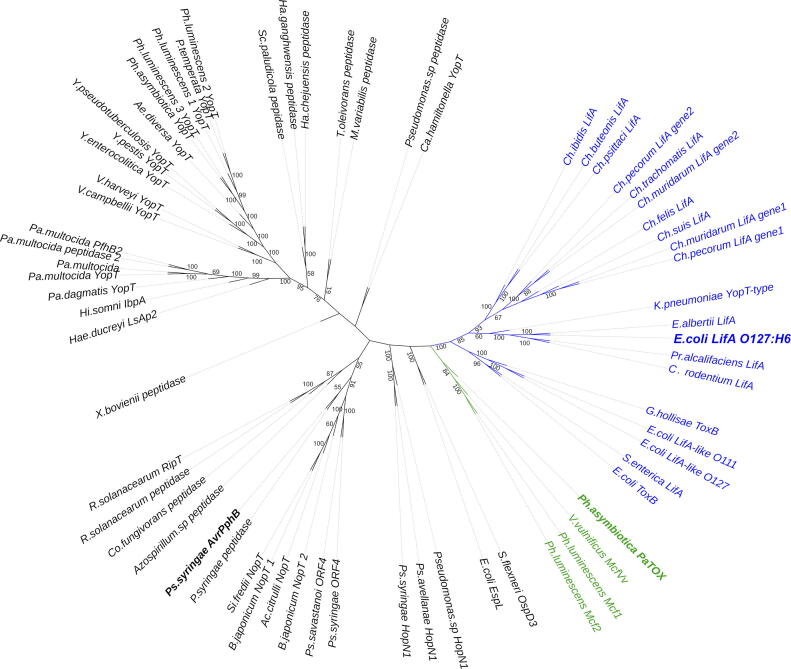
Lymphostatin shares sequence conservation with C58 cysteine protease domains (CPDs). Partial sequence alignments of MEROPS class C58 CPDs, lymphostatin (blue and bold) and lymphostatin-like CPDs were assembled with PSI-BLAST and aligned with Psi-Coffee. Phylogenetic analysis of the aligned sequences was used to generate an unrooted tree. Results from 100 bootstrap events have been used to annotate the branches of the tree (> 50% support value). Atomic resolution structural information is available for AvrPphB from *Pseudomonas syringae* (PDB ID: **1UKF**) and PaTox from *Photorhabdus asymbiotica* (PDB ID: **6HV6**); shown in bold. LifA shares higher sequence identity with PaTox than AvrPphB. Sequence alignments, sequence accession numbers and annotation of each catalytic triad are available in Supporting Information.

**Figure 3:**
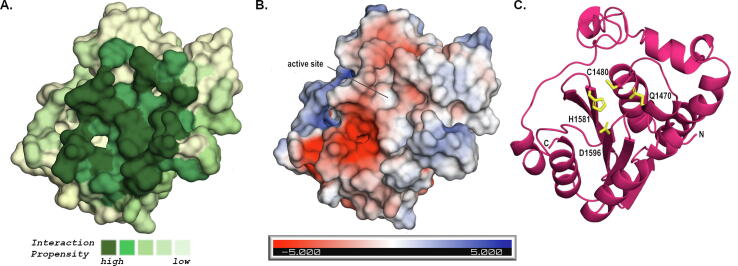
Lymphostatin has a C58 cysteine protease domain (CPD) with a putative papain-like fold. (a) A homology model of the C58 CPD of LifA based on PaTox (PDB ID: **6HV6**) constructed using the Phyre2 server.^30^ The protein surface has been coloured according to surface residue interaction propensity calculated using the docking program ArDock.^31^ The green surface represents the predicted enzyme active site. (b) Electrostatic surface representation of the lymphostatin CPD. The putative binding site forms a neutral groove (white) with a neighbouring negatively charged patch (red). (c) The CPD is displayed as a cartoon representation to highlight the papain-like fold predicted from sequence. The catalytic triad (C1480, H1581, D1596) and Q1470 are shown as yellow sticks. Q1470 is predicted to be placed to facilitate the formation of the canonical oxyanion hole necessary for the stabilisation of a negative charge on a reaction intermediate in cysteine proteases. The chain direction is indicated with N and C. All images have the same 3D orientation. Figures were prepared in PyMol (The PyMOL Molecular Graphics System, Version 2.4.0 Schrödinger, LLC).

To further investigate the proteolytic activity of lymphostatin we sought to better define the putative CPD. We assembled lymphostatin-like CPD sequences with PSI-BLAST^30^ and aligned them with Psi-Coffee (T-COFFEE, Version_11.00.d625267 (2016–01-11 15:25:41 - Revision d625267 - Build 507)).^31^ Phylogenetic analyses on the aligned sequences were used to generate an unrooted tree to guide our subsequent modelling^32^, ^33^, ^34^, ^35^, ^36^ (Figure 2). Lymphostatin and LifA-like proteins form a well-defined clade (blue). PaTox and the “makes caterpillars floppy” toxins Mcf1 and Mcf2 are grouped together (green).^37^ The majority of the YopT-like C58A subfamily, which includes YopT from *Yersinia pestis* form a clade as do the C58B subfamiliy that is rich in CPDs from bacterial plant pathogens (representative member AvrPphB from *Pseudomonas syringae)*. We included the *E.coli* protein EspL and its close homologue OspD3 from *Shigella flexneri* in our sequence alignments as they are proteins with proven cysteine protease activity found in the same pathogenicity island with other Type III secreted effectors. Sequence alignments and detailed alignment procedures are provided in the Supporting Information (Table S1 and Figures S1 and S2). Of the available atomic resolution structures, PaTox was the closest homologue and was used to build a 3D model with the Phyre2 server.^38^ The probability of the putative lymphostatin CPD (W1442–Q1619) being homologous to the PaTox C58-like CPD was 99.8%. Figure 3 shows the model of the putative lymphostatin CPD (W1442–Q1619) annotated to show the catalytic triad (C1480, H1581 and D1596) and Q1470. Q1470 is very well conserved in C58 CPDs and is positioned in space to facilitate the formation of the canonical oxyanion hole necessary for the stabilisation of a negative charge on a reaction intermediate. The active site is annotated on the electrostatic surface shown in Figure 3(B). The most likely binding interface was further defined using the docking program ArDock.^39^ It is interesting to note that most, but not all the surface residues predicted to have a high interaction propensity are very well conserved in LifA-like proteins (Figures S2 and S3(A)). This is best illustrated by the loop connecting α-helix-1 to α-helix-2 (V1474–Q1476) that lies immediately upstream of the catalytic cysteine motif: ^1477^[**E**,D]**G**[**R**,K]**C**[**M**,I,L,V,T]**G**^1482^. The position of the variable sequence close to the catalytic cysteine motif where it flanks the oxyanion hole would suggest that this sequence is key to substrate recognition. These data would indicate that lymphostatin forms a papain-like CPD.

### Inhibitors of endosome acidification reduce formation of the predicted cleavage product in a concentration-dependent manner

**Figure 4:**
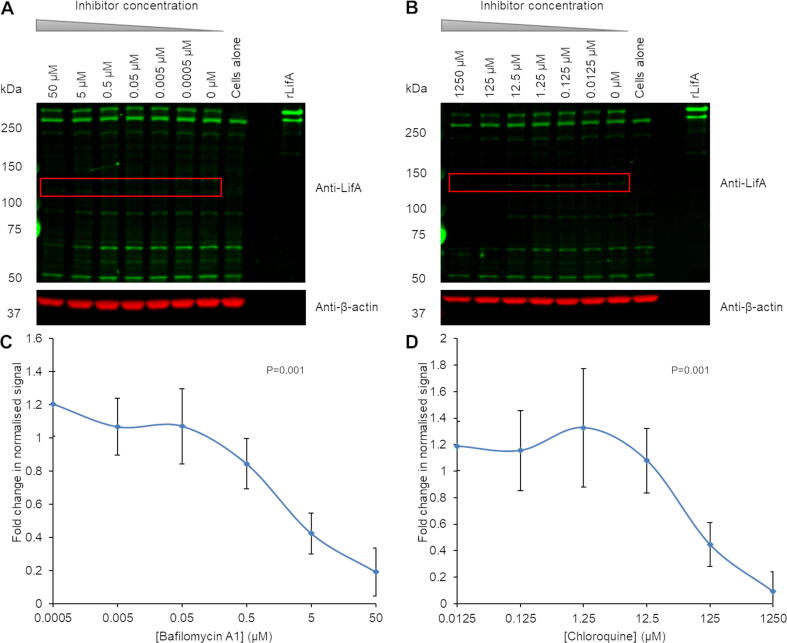
Inhibitors of endosomal acidification prevent lymphostatin cleavage. An absolute number of 1.2 × 10^7^ bovine T cells were incubated with the indicated final concentrations of bafilomycin A1 or chloroquine for 1 h then treated with WT rLifA for 1 h. A volume equivalent to 2 × 10^6^ cells was run in each well. (a) Western blot of bafilomycin-treated T cells shows that the appearance of the cleavage product (red box) is reduced with increasing inhibitor concentration; DMSO was used as the 0 µM control. (b) Western blot of chloroquine-treated T cells shows that presence of cleavage product remains constant between 0.0125 and 12.5 μM but is reduced at higher concentrations; PBS was used as the 0 µM control. Data were generated from 5 independent donors and a representative blot is shown. Quantification of signal by densitometry was performed on the western blots. The quantity of cleavage product at each inhibitor concentration was normalised against its respective actin signal to account for protein loading across the gel. Data were normalised again against the appropriate 0 µM controls to give a fold change in normalised signal compared to untreated cells. (c) Average fold change in normalised signal of bafilomycin-treated T cells with titration of inhibitor concentration. (d) Average fold change in normalised signal of chloroquine-treated T cells with titration of inhibitor concentration. Error bars indicate standard deviation of the average fold changes from 5 experiments.

Given that autocatalytic cleavage of the LCTs is dependent on acidification of the endosome, which induces a conformational change in the protein leading to insertion across the endosomal membrane, we investigated whether the processing of lymphostatin may be sensitive to bafilomycin A1 or chloroquine, which are known to block endosome acidification across diverse cell types and species. Bafilomycin A1 is a proton pump inhibitor,^40^ while chloroquine is a lysosomotropic agent that inhibits endosomal acidification through the prevention of lysosomal fusion.^41^ Separate treatment of bovine T cells with increasing concentrations of both inhibitors reduced the appearance of the 140 kDa species reactive to anti-LifA antibodies (Figure 4). Densitometry of the fluorescence intensity at this position (normalised to β-actin signals then expressed as fold-change relative to the diluent control over five independent replicates of the study) revealed that at increased concentrations of bafilomycin A1 and chloroquine, significantly lower quantities of the 140 kDa fragment are produced in comparison to the negative controls, even accounting for the loss of β-actin signal at higher concentrations that may be a consequence of cytotoxicity (Figure 4). Linear regression slopes across five donors for both bafilomycin A1 and chloroquine-treated cells were significantly different from the null hypothesis that inhibitor concentration has no effect on the presence of the 140 kDa cleavage product (*P* < 0.001). Endosome acidification was therefore inferred to be a requirement for the processing event.

### Mutation of C1480 of lymphostatin does not affect biophysical properties of the protein

**Figure 5:**
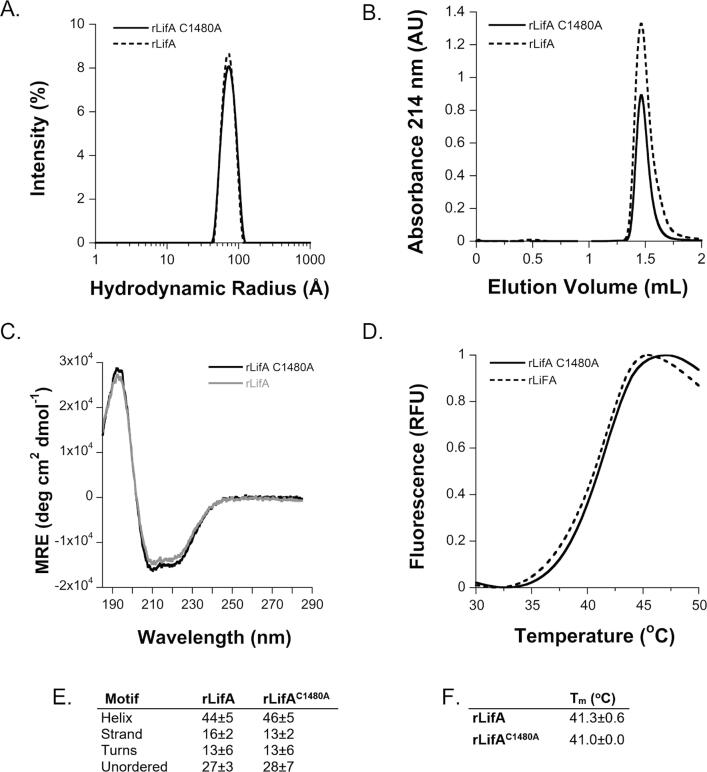
Biophysical analysis of rLifA^C1480A^. (a) rLifA^C1480A^ has the same hydrodynamic radius as WT rLifA; there was no evidence of aggregated protein in either sample. The size distribution by intensity of 1.1 µM rLifA^C1480A^ (R_h_ 78 ± 26 Å; mode and SD of the intensity distribution) compared to 1.1 µM WT rLifA (R_h_ 78 ± 29 Å; mode and SD of the intensity distribution), measured by dynamic light scattering. (b) Analytical size-exclusion chromatography of rLifA^C1480A^ and WT rLifA (Superose 6 Increase 3.2/300). (c) The CD spectra of 0.11 µM WT rLifA and rLifA^C1480A^. Far UV CD spectra expressed in mean residue ellipticity (MRE) as a function of wavelength. (d) WT rLifA and rLifA^C1480A^ thermal stability. The fluorescence of SYPRO Orange dye mixed with 0.1 μM WT rLifA and rLifA^C1480A^ was measured in triplicate over a temperature range of 15–70 °C to determine the *T*_m_ of each protein. A reproducible *T*_m_ of ~41 °C was observed for both proteins. (e) Secondary structural elements derived from the CD spectra of WT rLifA and rLifA^C1480A^. (f) *T*_m_ for WT rLifA and rLifA^C1480A^.

We know from previous studies that mutation of any of the three residues of the CHD catalytic triad abolishes activity of the CPD in LCTs and C58 proteases.^19^, ^42^, ^43^ Here, we elected to mutate C1480 in rLifA to a neutral alanine residue using site-directed mutagenesis. During purification, the resulting protein had similar purity, surface charge properties and hydrodynamic radius to WT rLifA and a DTD-AAA variant that we have also investigated^12^ (Figure S4(A)–(C)). To verify that the mutation did not grossly affect the folding and structure of the purified protein, we examined biophysical properties of the rLifA^C1480A^ protein and compared these to WT rLifA. We detected rLifA^C1480A^ at the same position as WT rLifA by gel electrophoresis and western blotting with anti-LifA antibodies (Figure S4(D)). The identity of full-length rLifA^C1480A^ was confirmed by in-gel protein digest and peptide mass fingerprinting. The peptide mass map generated was searched against the NCBInr database using the MASCOT search engine. Lymphostatin was identified as the top scoring hit with a MASCOT protein score of 485. Peptides aligned to 43% of the lymphostatin primary sequence; Glu60 to Arg3174 (of 3229), consistent with earlier analyses of the WT protein.^12^ rLifA^C1480A^ was found to be of a similar hydrodynamic radius to the monomeric WT protein by dynamic light scattering and eluted at the same position as WT rLifA in analytical size-exclusion chromatography (Figure 5(A) and (B)). rLifA^C1480A^ eluted as a single peak with an elution volume of 1.46 ± 0.01 mL, whereas monomeric rLifA protein eluted at 1.47 ± 0.01 mL (Superose 6 Increase 3.2/300, GE Healthcare). Analysis of secondary structure of WT rLifA and rLifA^C1480A^ by circular dichroism spectroscopy revealed a near-identical spectrum, showing similar α-helical content (approximately 44% WT rLifA and 46% rLifA^C1480A^), as well as β-strand content (approximately 16% WT rLifA and 13% rLifA^C1480A^, Figure 5(C) and (E)). Subtle changes to the secondary or tertiary structure of a protein can influence the Gibbs free energy of unfolding and its associated mid-point melting transition (*T*_m_).^44^, ^45^ We therefore used thermal denaturation fluorescence assays to measure the stability of the protein structure, which yielded a *T*_m_ of 41 °C and almost indistinguishable melting curves (Figure 5(D) and (F)). Taken together, these assays indicate that the mutant protein is near-identical in secondary and tertiary structure to WT lymphostatin.

**Figure 6:**
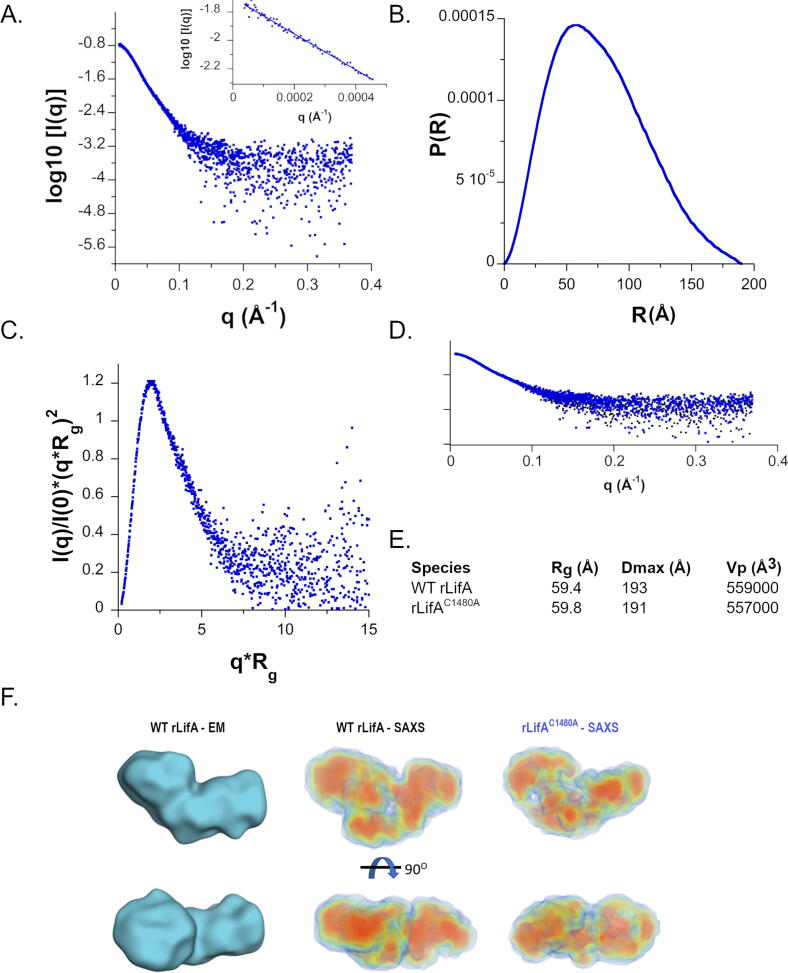
SAXS analysis of rLifA^C1480A^. (a) The experimental SAXS scattering curve of rLifA^C1480A^. The logarithm of the SAXS intensity *versus* the scattering vector, q (Å^−1^). The linear region of the Guinier plot (ln[I(q)] *versus* q^2^) is shown as an inset. (b) The Pair-distance distribution function of rLifA^C1480A^. (c) The dimensionless Kratky plot (I(q)/I(0)*(q*R_g_)^2^) of rLifA^C1480A^ is consistent with that of a globular protein. (d) A comparison of the scattering curves of WT rLifA and rLifA^C1480A^ normalised to I(0). (e) WT rLifA and rLifA^C1480A^ have very similar geometric parameters. (f) Negative stain electron microscopy envelope of WT rLifA (blue) reported in our previous publication.^10^ Electron density map generated from solution scattering using DENSS for rLifA^C1480A^ and WT rLifA shown in two views related by a 90° rotation around the horizontal axis. Maps are shown as volumes and coloured according to density; red > orange > yellow > green > blue.

To compare the three-dimensional structure of rLifA^C1480A^ to WT rLifA, we undertook a SAXS analysis of rLifA^C1480A^ coupled to size-exclusion chromatography. Transformation of the scattering data gave a linear Guinier region indicative of an absence of interparticle effects; R_g_ 58.9 ± 1.7 Å (Figure 6(A)). The real space pair distribution function reflects the scattering from a slightly elongated globular particle (Figure 6(B)). The longest particle dimension D_max_ is 191 Å. The real space R_g_ 59.8 ± 0.0 Å calculated from the P(r) function is consistent with the result from Guinier analysis which strengthens the validity of the solution. Kratky analysis shows an almost bell-shaped curve that converges to the q axis with increasing q (Figure 6(C)). The peak maxima for rLifA^C1480A^ is close to the Kratky-Guinier point, the peak position for an ideal globular protein. The Porod volume (V_p_) was determined to be 557285 Å^3^. These data are essentially identical to SEC-SAXS data collected for WT rLifA; D_max_ is 193 Å, R_g(Guinier)_ 58.7 ± 1.2 Å, R_g(real)_ 59.4 ± 2.0 Å V_p_ 559196 Å^3^ purified under the same conditions as rLifA^C1480A^ as part of this study (Figure 6(D)).

We undertook *ab initio* shape determination to compare the particle envelope of rLifA^C1480A^ to WT protein. Lymphostatin is a large multi-domain protein that has proved recalcitrant to crystallographic study. Kratky analysis of 1D scattering profiles (Figure 6(C)) and preliminary cryo-electron diffraction experiments suggest that there is some flexibility within this large molecule in solution. Traditional bead modelling algorithms generate models with no indication of electron density within the particle envelope. Moreover, for flexible molecules generating a large number of bead models, averaging them and filtering to retain a specific bead of high probability invariably produces an envelope with an artefactually small molecular envelope that excludes extended flexible regions. To better investigate variations in electron density within lymphostatin we calculated 3D electron density maps from the solution scattering data using the program DENSS (DENsity from Solution Scattering) which employs iterative phase and structure factor retrieval.^46^

A comparison of the predicted shape and dimensions of rLifA^C1480A^ and rLifA obtained by SAXS and EM (Figure 6(F)) support the notion that rLifA^C1480A^ has a very similar architecture to the WT protein.^12^ We cannot preclude the possibility that the C1480A substitution causes small conformational changes that would not be detected at this resolution. To further validate our electron density maps, we fitted our homology models of the glycosyltransferase domain and CPD of lymphostatin to the maps, basing the domain orientation on the crystal structure of the homologous TcdA from *Clostridium difficile* (PDB ID: **4R04**, Figure S5). The domain arrangement both fits well in terms of putative electron density and exposes the active sites of the glycosyltransferase and cysteine protease domains to solvent where there is an access route for a substrate. We hypothesise that the largest lobe of our *ab initio* shape determination envelope represents the glycosyltransferase domain. This is consistent with SAXS data we have obtained for fragments of lymphostatin (data not shown).

LCTs undergo a conformational change and autocatalytic cleavage upon on acidification of the endosome. To investigate the effect of pH upon the conformation of lymphostatin we repeated the SEC-SAXS analysis at pH 5.5. We have consistently observed that WT rLifA and rLifA^C1480A^ elute later in size-exclusion chromatography under acidic conditions (Table S2). This represents a change in stokes radius/hydrodynamic character. Analysis of the scattering data confirmed both rLifA and rLifA^C1480A^ remain monomeric in solution at pH 5.5 and that there was no evidence of protein aggregation. R_g_ and D_max_ at pH 5.5 and pH 7.5 are indistinguishable for both rLifA and rLifA^C1480A^. The Porod volume was slightly larger and the Porod exponent smaller at pH 5.5 compared to pH 7.5 for both WT and mutant proteins. A smaller Porod exponent is indicative of a more flexible system. The electron density maps generated for WT rLifA and rLifA^C1480A^ at pH 5.5 occupy similar volumes, but there does appear to be slightly less well defined centres of electron density (Figure S6). We conclude that the change from neutral to acidic pH does not induce any gross structural changes, but results in a small increase in flexibility under acidic conditions.

### Mutation of C1480 of lymphostatin abolishes the appearance of the 140 kDa sub-fragment

**Figure 7:**
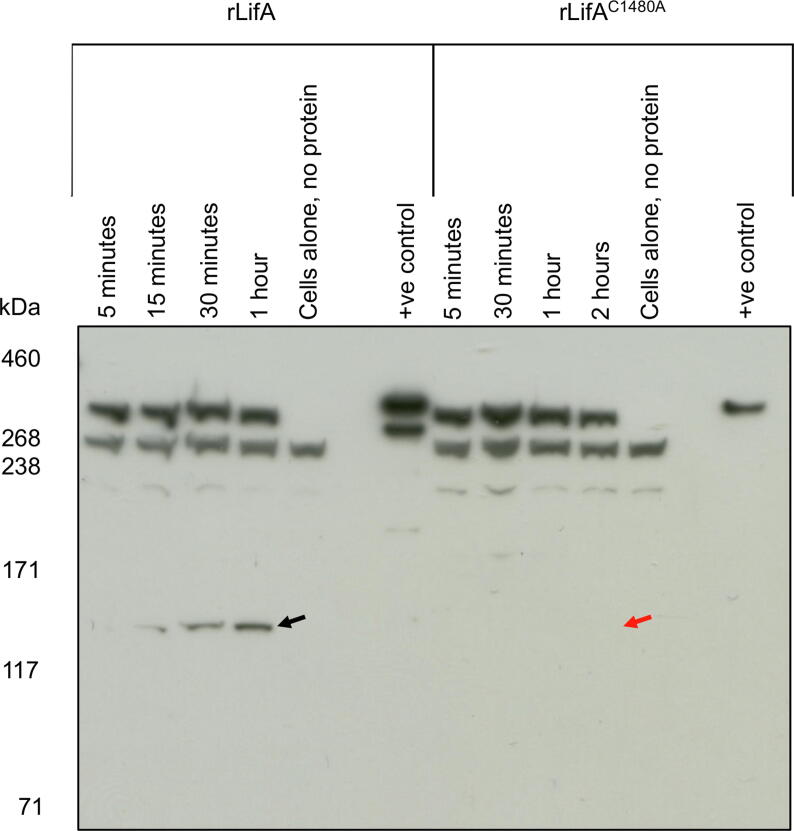
Cleavage of lymphostatin in T cells is dependent on the cysteine protease motif. Western blot of lysates from T cells (1.2 × 10^7^ cells) treated with rLifA and rLifA^C1480A^ for varying durations. An approximately 140 kDa cleavage product of rLifA is barely visible at 5 min post-incubation and increases in concentration up to 1 h after treatment (marked with black arrow), while no cleavage product is observed in LifA^C1480A^ treated T cells even after 2 h (red arrow indicates where cleavage product would be expected to be observed). Quantities of 2 ng of rLifA or rLifA^C1480A^ were used as positive (+ve) controls. Data were generated from 2 independent donors and a blot is shown from a representative donor.

In T cells treated with the WT rLifA protein, the approximately 140 kDa species reactive with anti-LifA antibodies was clearly evident by 15 min after addition of the protein, and the signal intensity increased up to 1 h post-addition (Figure 7). In contrast, the 140 kDa protein was not observed in the lysates of T cells treated with rLifA^C1480A^ at any time post-addition (Figure 7). As phenotypes attributable to LifA have been described in other cell types, including inhibition of mitogen-activated IL-2 synthesis in Jurkat cells^9^ and pedestal formation on epithelial cells,^15^ we examined if processing of LifA could be detected in J774A.1 murine macrophage-like cells. In J774A.1 cells treated with WT rLifA protein, both the approximately 140 kDa and the approximately 225 kDa species reactive to anti-LifA antibodies could be detected after 1 h (Figure S7(A)). In contrast, these proteins were not observed in the lysates of J774A.1 cells treated with rLifA^C1480A^ (Figure S7). Additionally, the 225 kDa species was reactive to anti-6 × His antibody, indicating that it is the C-terminal portion of lymphostatin (Figure S7(B)).

### The C1480A substitution abolishes lymphostatin activity

**Figure 8:**
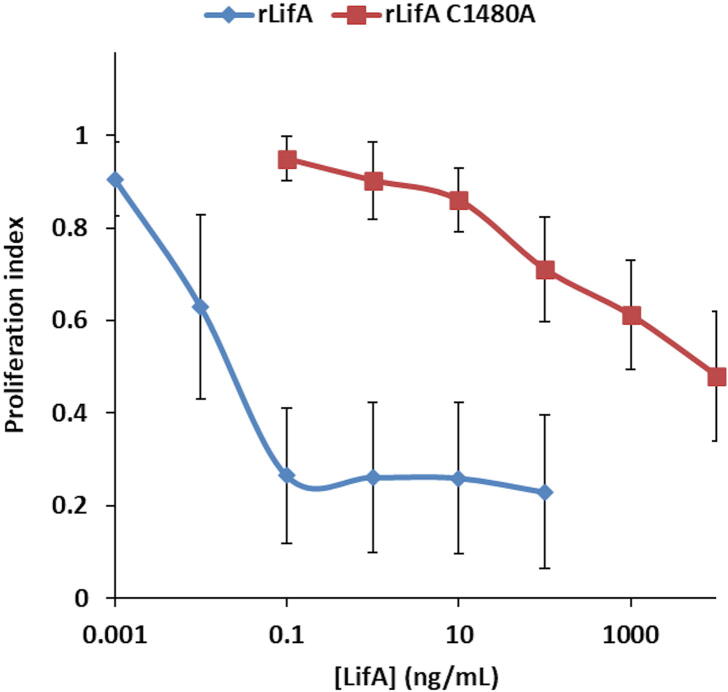
The C1480A substitution abolishes inhibitory activity of lymphostatin. Effect of concentration titration of WT rLifA and rLifA^C1480A^ against ConA-stimulated peripheral bovine T lymphocytes. An absolute number of 200,000 cells were seeded into the wells of a 96 well plate. Titrations were carried out in triplicate per donor and the results are the average obtained from 5 independent experiments using separate donors. Data were normalised against cells with ConA alone to give a ratio index of proliferation. Error bars indicate the standard deviation of the average ratios from across the 5 experiments. The calculated ED_50_ was 0.014 ± 0.004 ng/mL (WT rLifA) and 1215 ± 684 ng/mL (rLifA^C1480^). ED_50_ Values for each donor can be found in Table S3.

Purified lymphostatin is a potent inhibitor of the mitogen- and antigen-stimulated proliferation of bovine T cells, including all key subsets.^11^, ^12^ We therefore investigated whether the C1480A substitution affected this activity. Using T cells from five independent donors, rLifA^C1480A^ was shown to have a markedly reduced ability to inhibit ConA-stimulated T cell proliferation in comparison to WT rLifA (Figure 8). The 50% effective dose (ED_50_) of rLifA^C1480A^ was 5 orders of magnitude higher than that of WT rLifA (ED_50_ WT rLifA = 0.014 ± 0.004 ng/mL, ED_50_ rLifA^C1480A^ = 1215 ± 684 ng/mL). This increase in ED_50_ was highly significant across five donors (*P* = 0.0097) and was comparable to that seen previously for lymphostatin with a substitution of three alanine residues for a DTD motif in the glycosyltransferase domain (rLifA^DTD-AAA^).^12^ Our data indicate that the cysteine protease motif is required for the ability of lymphostatin to block mitogen-activated proliferation of bovine T cells, without obvious alteration of its secondary or tertiary structure.

### Concentration-dependent inhibition of concanavalin A-activated proliferation of bovine T cells by wild-type lymphostatin is not associated with direct cytotoxicity

To confirm that lymphostatin is not associated with direct cytotoxicity we measured the release of lactate dehydrogenase (LDH). Three replicates of the proliferation assays were set up in parallel, one to be used for measurement of the ability of LifA to inhibit cellular proliferation and two to be used for measurement of LifA’s cytotoxicity at time intervals of 24 and 72 h post-treatment (Figure S8). At 24 h post-treatment, percent cytotoxicity was largely unchanged across the LifA concentration range, indicating that the effect on lymphocyte proliferation is not associated with direct cytotoxicity (Figure S8(B)). At 72 h post-treatment, at concentrations of LifA insufficient to block proliferation, the lymphocytes are driven to exhaustion by the mitogen resulting in significant LDH release (Figure S8(C)). However, at concentrations where LifA achieves saturating inhibition of lymphocyte proliferation ( 0.1 µg/mL and above), negligible LDH release was observed.

## Discussion

Elucidation of the molecular mechanisms underlying microbial pathogenesis can inform the design of strategies to control infections and yield insights into host cell biology. Here we characterised the putative cysteine protease motif of lymphostatin, a virulence factor produced by attaching & effacing *E. coli*. Although we know from previous studies that the activity of lymphostatin is dependent on the presence of a glycosyltransferase motif,^12^ there has been little investigation of the role of other recognised motifs within lymphostatin, including the cysteine protease domain which mediates autocatalytic processing of the structurally homologous large clostridial toxins.^42^, ^43^

By western blotting of lymphostatin-treated bovine T cells, we observed the appearance of a single specific anti-LifA-reactive species of approximately 140 kDa, consistent with expectations for the glycosyltransferase domain of LifA and autocatalytic cleavage at a site N-terminal to the cysteine protease motif, as described for LCTs.^27^, ^28^, ^29^ We were unable to observe the predicted 225 kDa species representing the predicted C-terminal portion of lymphostatin in T cells, however, this was detected in J774A.1 cells treated with rLifA. It is possible that this reflects differences in the rate of uptake and processing of rLifA, variation in the rate of retention and degradation of the 225 kDa fragment in endosomes, and/or the limit of detection of the assay used. Additionally, since we observed non-specific reactivity of the antibody with T cell proteins at around the same size, this may have obscured the 225 kDa species. As autocatalytic cleavage of LCTs by the cysteine protease motif requires inositol hexakisphosphate as a co-factor,^21^, ^27^, ^28^, ^29^ we attempted to detect processing of WT rLifA in the presence of this molecule *in vitro*. However, we were unable to observe specific processing of the protein, albeit further optimisation of the assay conditions may be required.^12^

We also attempted to determine whether lymphostatin is capable of acting as a protease against host proteins similar to other C58 proteases.^19^, ^47^ Even if the cysteine protease motif is responsible for autocatalytic cleavage, it may also cleave host proteins, as observed for AvrPphB, a C58 protease produced by *P. syringae*. In AvrPphB the cysteine protease motif is required for autocatalytic cleavage of the protein to expose a myristoylation site and also the cleavage of the host cell protein kinase PBS1.^19^, ^47^, ^48^ In an assay using release of fluorescein-conjugated peptides from labelled casein as a model host substrate cleaved by a variety of proteases, we were unable to detect protease activity of WT rLifA *in vitro*, at least under the conditions tested (see Footnotes).

The appearance of 140 and 225 kDa proteins detectable by anti-LifA antibody in a manner dependent on residue C1480 is consistent with autocatalytic cleavage by a mechanism similar to LCTs. As a result, it would be desirable to confirm the location of the cleavage site that produces these two fragments. We were unable to detect LifA-derived tryptic peptides from gel slices of electrophoresed lysates of T cells treated with WT rLifA at 140 kDa by mass spectrometry, likely as the species is present at very low abundance in an excess of lymphocyte proteins. Confirmation of the cleavage site would likely require immunoprecipitation of the fragments from a large quantity of rLifA-treated T or J774A.1 cells. We anticipate that the 140 kDa fragment encompasses the N-terminal glycosyltransferase domain required for lymphostatin activity. In line with this, we further hypothesise that this fragment acts on an intracellular target after processing and that the C-terminal fragment is responsible for binding and uptake of lymphostatin. We sought to test the activity of the N-terminal fragment by direct delivery of a rLifA fragment encompassing residues 1–1620 including the glycosyltransferase domain (informed by limited proteolysis of rLifA)^12^ to the cytoplasm. Unfortunately, these studies were impeded by the fact that the protein transfection reagent used was able to activate primary T cells (see Footnotes).

The appearance of the 140 kDa fragment of rLifA was shown to be dependent on endosomal acidification using a proton pump inhibitor (bafilomycin A1) and a lysosomotropic agent (chloroquine). Treating cells with increasing amounts of the inhibitors poisons the cells, such that it is not possible to titrate the appearance of the fragment to, or close to, zero. Better understanding the effect of pH on processing of lymphostatin would be more controlled in an *in vitro* biochemical assay, however, this is dependent on identifying any co-factor that is required, which so far has been elusive.

We have observed small but consistent changes in the flexibility of rLifA and rLifA^C1480A^ and a concomitant change in the volume of the molecular envelope in acidic buffers. Increasing protein flexibility means that in acidic conditions lymphostatin samples new conformations. This change in flexibility is not sufficient to activate autocatalytic cleavage *in vitro* but may well expose a co-factor binding site or remodel part of the protein to facilitate membrane insertion in host cells.

The C1480A substitution herein represents a less intrusive change to the lymphostatin protein than has previously been reported using in-frame deletions that encompass the CHD catalytic triad (residues 1478–1600^6^ or residues 1453–1480.^7^, ^26^ The impact of these deletion mutations has been reported, for example a loss of lymphostatin activity^6^ and impacts on E-cadherin and β-catenin at apical enterocyte junctions.^26^ However, in these studies it was unclear if indirect effects of the mutations on secondary or tertiary structure of the protein may have occurred. Our biophysical measurements suggest minimal impact of the C1480A substitution, and consequently we infer that the observed impact of lymphostatin activity reflects the role of the cysteine protease domain *per se*.

## Materials and Methods

### Site-directed mutagenesis of LifA

The cysteine residue at amino acid position 1480 of full-length lymphostatin from the prototype EPEC O127:H6 strain E2348/69 was mutated to an alanine residue by site-directed mutagenesis (QuikChange II XL, Agilent Technologies) according to the manufacturer’s instructions. Our previously described construct for rhamnose-inducible expression of a histidine-tagged version of LifA was used as a template (pRHAM-LifA-6xH)^12^ and the primers LifA-C1480A-1 (forward), 5′CTATTGGTAACCCAAGAAGGACGC**GCA**ATGGGATTAGCCTTACTTTATT3′, and LifA-C1480A-2 (reverse), 5′TAAATAAAGTAAGGCTAATCCCAT**TGC**GCGTCCTTCTTGGGTTACCAATAG3′, were used to introduce the mutation. For ease of screening, a *BsrD*I restriction site (underlined) was introduced to the sequence at the site of the C1480A substitution (indicated in bold). Putative plasmid clones were screened for the substitution by *BsrD*I digestion of amplicons spanning the target sequence and candidate mutants were analysed by Sanger sequencing of both strands (GATC Biotech and Edinburgh Genomics). A clone with 100% nucleotide identity to the native *lifA* sequence over the full length of the gene, except at the site of C1480A substitution, was selected.

### Expression and purification of recombinant LifA^C1480A^

Recombinant His-tagged lymphostatin harbouring the C1480A substitution (rLifA^C1480A^) was overexpressed and purified using the same method previously described for WT rLifA and a DTD-AAA variant (Figure S4).^12^ WT rLifA was purified using an existing expression clone^12^ by the same method with near identical purification characteristics. The presence of lymphostatin after expression and at each purification step was monitored by SDS-PAGE and either Coomassie staining of 4–12% Tris-glycine gels or western blotting of 3–8% Tris-acetate gels with a rabbit polyclonal anti-LifA antibody, clone 45 (generated by Dundee Cell Products using purified full-length WT rLifA protein). Membranes were blocked with Odyssey Blocking buffer in TBS (LI-COR Biosciences) for 1 h at room temperature on a rocker. Membranes were washed three times with TSB-T (TBS with 0.1% (v/v) Tween 20 (VWR International)) then anti-LifA pAb serum was added to membranes at a dilution of 1/20,000 in TBS-T and incubated a 4 °C overnight on a rocker. Membranes were washed twice with TBS-T, with a third wash using PBS-T (PBS with 0.1% Tween 20). Bound antibody was detected using a goat anti-rabbit IgG antibody conjugated to DyLight™ 800 (green) 4X polyethylene glycol-conjugated fluorescent dye (Cell Signaling Technology) diluted 1/30,000 in PBS-T and incubated at room temperature for 1 hour on a rocker. Blots were washed three times with PBS-T then imaged using a LI-COR Odyssey Imaging System (LI-COR Biosciences).

### Analytical size-exclusion chromatography

Analytical size-exclusion chromatography was performed on the purified WT rLifA and rLifA^C1480A^ for comparison. A volume of 25 µL of 0.11 µM lymphostatin was loaded onto a Superose 6 Increase 3.2/300 size-exclusion chromatography column (GE Healthcare) pre-equilibrated in 50 mM Bis-Tris, pH 7.5, 100 mM NaCl, 3% (v/v) glycerol at a flow rate of 0.05 mL/min on an ÄKTAmicro chromatography system (GE Healthcare).

### Dynamic light scattering

WT rLifA and rLifA^C1480A^ were exchanged into Assay Buffer (10 mM Tris, pH 7.6, 150 mM NaCl) using a HiTrap desalt column (GE Healthcare) at a flow rate of 4 mL/min, and centrifuged at 12,000***g***, 4 °C for 15 min to remove any dust particles that might interfere with size estimation. The mean hydrodynamic radius of a 1.1 µM solution of lymphostatin was measured using a Zetasizer Automated Plate Sampler (Malvern Instruments, UK) equipped with a 50 mW laser light source, with a wavelength of 830 nm. Data were collected at a scattering angle of 90° at 25 °C in 10 s accumulations repeated 13 times. The average autocorrelation data was fitted to a model optimised for protein solutions using the software supplied with the instrument. This analysis generated a distribution of particles by size.^49^ Experiments were repeated five times.

### Matrix assisted laser-desorption ionisation (MALDI) mass spectrometry

Excised SDS-PAGE gel-bands of rLifA^C1480A^ were incubated in 50 mM ammonium bicarbonate containing porcine trypsin (Promega) in a trypsin:lymphostatin ratio of ~1:30, overnight at 32 °C. Peptides were identified by MALDI mass spectroscopy on an ultrafleXtreme™ mass spectrometer (Bruker) using an α-cyano-4-hydroxycinnamic acid matrix. A peptide mass map was generated from spectral data using Compass DataAnalysis 4.4 software (Bruker). Peptide masses were searched against the National Center for Biotechnology Information database of non-identical protein sequences (NCBInr) with the MASCOT search engine,^50^ mass tolerance of 10 ppm (Matrix Science). Peptide masses were also compared to the sequence of full-length LifA from EPEC E2348/69 (Protein Prospector software).^12^, ^13^, ^51^ The instrument was calibrated prior to each data acquisition and an internal calibration performed on the digest products of porcine trypsin.

### Circular dichroism (CD)

The far ultraviolet (UV) CD spectra of 0.11 µM WT rLifA and rLifA^C1480A^ were recorded at 10 nm/min; data pitch 0.1 nm; response time 2 s between 185 and 285 nm in a 0.1 cm path length quartz cuvette at 25 °C (Jasco-810 spectropolarimeter). The proteins were exchanged into 10 mM NaH_2_PO_4_, pH 7.6, 100 mM NaF prior to analysis using a HiTrap desalt column (GE Healthcare) at a flow rate of 4 mL/min. Spectra were corrected by subtracting a buffer baseline, each an average of five spectra, acquired under the same conditions. Secondary structure was estimated using the DichroWeb CD secondary structure analysis server^52^ including the methods CONTIN, SELCON3 and CDSSTR^53^, ^54^, ^55^, ^56^ and reference data sets 3, 4, 6, 7, SP175 and SMP180.

### Thermal denaturation fluorescence assays

The temperature-induced unfolding of lymphostatin was followed using the environmentally sensitive dye SYPRO Orange (Invitrogen). SYPRO Orange shows an increase in fluorescence correlated with the exposure of hydrophobic residues upon unfolding.^44^, ^45^ The fluorescence of WT rLifA and rLifA^C1480A^ (both 0.1 µM) in 5 × SYPRO Orange was measured between 15 and 70 °C at 0.5 °C increments every 30 s (Bio-Rad IQ5 Multicolor Real-Time PCR Detection System). The proteins were exchanged into Assay Buffer prior to analysis using a HiTrap desalt column (GE Healthcare) at a flow rate of 4 mL/min. Relative fluorescence units (RFU) for each sample were plotted against temperature and the unfolding transition temperature (*T*_m_) of each protein was calculated from the steepest part of the melting curve. Experiments were repeated in triplicate.

### Small angle X-ray scattering coupled to size-exclusion chromatography (SEC-SAXS)

Small angle X-ray scattering (SAXS) measurements were carried out on beamline B21 at Diamond Light Source, Didcot, UK; Experiment No. SM18931-1; fixed camera length configuration (4.014 meters; useful q range 0.0031 to 0.38 Å^−1^); 12.4 keV beam. A volume of 45 µL of 2 mg/mL rLifA^C1480A^ was loaded onto a Superose 6 Increase 3.2/300 size-exclusion chromatography column (GE Healthcare) pre-equilibrated in 50 mM Bis-Tris, pH 7.5, 100 mM NaCl, 3% (v/v) glycerol; 15 °C at a flow rate of 0.075 mL/min using an Agilent 1200 HPLC system. Small angle X-ray scattering was collected on the sample as it eluted from the column in 10 s acquisition blocks at 15 °C. SAXS data reduction was performed in the Diamond Light Source software pipeline; ScÅtter (v3.1v).^57^ Analysis of SEC-SAXS data segments was carried out in ScÅtter (http://www.bioisis.net/scatter). Simple geometric parameters were calculated using ScÅtter, PRIMUS and the ATSAS 2.8.34 suite of programs.^58^ DENSS was run 20 times in the slow mode, each time with a different random seed. The maps fitting the data (19 maps in total), were aligned with automated enantiomer generation and selection, averaged, and refined. Electron density maps are shown as volumes and coloured according to density; red > orange > yellow > green > blue. Figures were drawn in the molecular graphics program USCF Chimera^59^ and PyMol (The PyMOL Molecular Graphics System, Version 2.4.0 Schrödinger, LLC).

### Isolation of peripheral blood mononuclear cells and T lymphocytes from bovine blood

Animal use was approved by the local Animal Welfare and Ethical Review Board and venous blood was collected from 12 to 18-month-old Holstein-Friesian cows (200 mL per donor per sampling day) in full compliance with requirements of the Animals (Scientific Procedures) Act 1986. Peripheral blood mononuclear cells were isolated and T lymphocytes subsequently enriched using sterile wool columns (Park Scientific) as previously described.^12^ T cell enrichment was monitored by flow cytometry of cells stained with anti-CD3 antibody as reported^12^ and the average purity of T cells achieved was 74.3% (54.9–90%). All samples were analysed with single channel staining on an LSR Fortessa X20 using CellQuest (BD Biosciences) and FlowJo v10 software (Tree Star). A minimum of 10,000 events were collected with an initial gate for live cells based on forward/side scatter parameters.

### Analysis of lymphostatin processing in T cells

Enriched peripheral bovine T cells (1.2 × 10^7^ cells) were incubated in serum-free Roswell Park Memorial Institute (RPMI-1640) medium with 25 mM HEPES (Sigma). After 1 h, WT rLifA or rLifA^C1480A^ was added to the cells at a final concentration of 2 µg/mL and incubated at 37 °C for the times indicated. Cells were washed twice with ice-cold phosphate-buffered saline (PBS), pelleting the cells in between by centrifugation at 17,949***g***, 4 °C for 1 min. As free lymphostatin sticks to plastic, the cells were transferred to clean microcentrifuge tubes after each wash. Cells (1.33 × 10^8^ cells/mL) were lysed with 90 µL of NP-40 lysis buffer (20 mM Tris, pH 7.4, 150 mM NaCl, 2 mM EGTA, 50 mM NaF, 1% (v/v) NP-40, 1 mM EDTA, 1 mM sodium orthovanadate, 1 cO™mplete mini protease inhibitor tablet/10 mL) and incubated on ice for 30 min. Insoluble debris was removed by centrifugation (17,949***g***, 4 °C for 10 min). Supernatants were denatured at 70 °C with 1 × NuPAGE sample reducing agent and 1 × NuPAGE LDS sample buffer (Invitrogen) for 10 min and stored at −20 °C until required. Western blots were performed using rabbit polyclonal anti-LifA as described above with 5% bovine serum albumin (Merck) in TBS-T used as an initial blocking agent. All washes were performed with TBS-T. Bound antibody was detected using a goat anti-rabbit IgG conjugated to horseradish peroxidase (Bio-Rad) diluted 1/10,000 with 2% (w/v) skimmed milk powder (Chem Cruz) as a blocking agent. HRP-conjugated signals were captured using SuperSignal™ (Thermo Scientific) and Hyperfilm ECL autoradiography film (GE Healthcare Life Sciences).

### Analysis of lymphostatin processing in J774A.1 cells

J774A.1 murine macrophage-like cells (1 × 10^6^ cells/well; ATCC) were seeded into 12 well flat bottom plates (Costar) for 24 h then incubated in serum-free RPMI-1640 medium with 25 mM HEPES. After 1 h, WT rLifA or rLifA^C1480A^ was added to the cells at a final concentration of 1 µg/mL and incubated at 37 °C for 1 h. Cells (8.33 × 10^6^ cells/mL) were lysed with 120 µL of NP-40 lysis buffer and processed as described above. Western blots were performed using rabbit polyclonal anti-LifA or mouse monoclonal anti-6 × His (BioLegend) as described above. Bound antibody was detected using a goat anti-rabbit IgG antibody conjugated to DyLight™ 800 (green) 4X polyethylene glycol-conjugated fluorescent dye or anti-mouse IgG conjugated to DyLight™ 800 (green) fluorescent dye using a LI-COR Odyssey Imaging System (LI-COR Biosciences).

### Inhibition of endosome acidification

To examine the effects of inhibitors of endosomal acidification on lymphostatin cleavage, T cells were incubated in serum-free RPMI medium supplemented with 25 mM HEPES. The inhibitors were added at a range of concentrations with bafilomycin A1 (50–0.0005 µM, LC Laboratories) or chloroquine diphosphate (1250–0.0125 µM, Sigma) informed by previous studies.^60^, ^61^ Dimethylsulphoxide (DMSO) and PBS were used as carrier controls, respectively. Fluorescent western blot analysis was used to detect LifA or predicted cleavage products thereof, and β-actin was detected as an internal standard using mouse anti-bovine actin (GeneTex) detected with goat anti-mouse IgG conjugated to DyLight™ 680 (red) fluorescent dye (Cell Signaling Technology). Blots were scanned using a LI-COR Odyssey Imaging System (LI-COR Biosciences), and densitometry was performed using Image Studio Lite v5.2 (LI-COR Biosciences). Densitometry was performed by first normalising the fluorescent signal of the lymphostatin cleavage product at each inhibitor concentration to its respective β-actin control. Data were normalised again to the appropriate carrier control to give fold changes in normalised fluorescent signal.

### Lymphocyte proliferation assay

Enriched bovine T cells were used to test the ability of WT rLifA and rLifA^C1480A^ to inhibit the mitogen-activated proliferation of lymphocytes as previously described.^12^ Briefly, cells were plated at 2 × 10^5^ cells/well in 96 well flat bottom plates (Costar) in triplicate for all conditions. Proteins were added at the concentrations as indicated. Cell proliferation was stimulated using ConA (Sigma) at a final concentration of 1 µg/mL in the presence or absence of WT rLifA or rLifA^C1480A^ at the concentrations indicated in a final volume of 100 µL/well. Cells were incubated at 37 °C for 72 h. The colorimetric substrate CellTiter 96® AQueous One (Promega) was added 18 h before the end of the assay. All measurements were carried out at 492 nm on a Multiskan Ascent plate reader (Thermo Scientific). Cells and medium alone were used as negative controls. Background medium measurements were subtracted from all values. All treatments are expressed as a Proliferation Index calculated as the ratio of Absorbance (Cells treated with ConA and recombinant protein)/Absorbance (Cells treated with ConA alone).

### Cytotoxicity assay

Cytotoxicity assays were performed in parallel with proliferation assays, using T cells purified as described above, from the same batch from three separate donors. Three replicates of the proliferation assays were set up in parallel, one to be used for measurement of the ability of LifA to inhibit cellular proliferation and two to be used for measurement of LifA’s cytotoxicity. At 24 and 72 h post-incubation, cytotoxicity was measured via an LDH release assay, using the Cytotoxicity Detection KitPlus (LDH) (Roche, UK), following manufacturer’s instructions. Cytotoxicity was expressed as a percent cytotoxicity, calculated as (Absorbance (cells treated with ConA and recombinant LifA) − Absorbance (untreated cells))/(Absorbance (positive control cells) − Absorbance (untreated cells)) × 100.

### Statistical analyses

Calculation of the effective dose of WT rLifA and rLifA^C1480A^ to inhibit T cell proliferation by 50% (ED50) was determined using drc in R.^32^, ^62^ The differences between ED_50_ values for rLifA and rLifA^C1480A^ were calculated for each donor then transformed by log10. A one sample T-test was used to determine whether the difference in ED_50_s, transformed by log10, were statistically significantly different. Linear regression analysis was carried out for the endosomal inhibitor experiments using Minitab 17.^63^ Average regression slopes were tested for statistical significance using a one-sample *T*-*test*. *P* values ≤ 0.05 were taken to be statistically significant.

### Accession numbers

Small angle scattering data and structural models have been deposited at the Small Angle Scattering Biological Data Bank (SASBDB) (https://www.sasbdb.org/) with the following accession numbers: **SASDKL9** (WT rLifA pH7.5), **SASDKM9** (WT rLifA pH5.5), **SASDKN9** (rLifA^C1480A^ pH7.5), **SASDKP9** (rLifA^C1480A^ pH5.5).

## Footnotes

Bease, A. G. (2019) Mode of action of a novel lymphocyte inhibitory factor of attaching & effacing *Escherichia coli*. *Doctor of Philosophy Thesis*, The University of Edinburgh.

## CRediT authorship contribution statement

**Andrew G. Bease:** Conceptualization, Methodology, Formal analysis, Investigation, Writing – original draft, Writing – review & editing. **Elizabeth A. Blackburn:** Conceptualization, Methodology, Formal analysis, Investigation, Writing – original draft, Writing – review & editing, Funding acquisition. **Cosmin Chintoan-Uta:** Methodology, Formal analysis, Investigation, Writing – review & editing. **Shaun Webb:** Methodology, Formal analysis, Investigation, Writing – review & editing. **Robin L. Cassady-Cain:** Conceptualization, Writing – original draft, Writing – review & editing. **Mark P. Stevens:** Conceptualization, Writing – review & editing, Supervision, Project administration, Funding acquisition.
