# Supplementary material for: Activity of Lymphostatin, A Lymphocyte Inhibitory Virulence Factor of Pathogenic Escherichia coli, is Dependent on a Cysteine Protease Motif
Source: J Mol Biol. 2021 Sep 17;433(19):167200. doi: 10.1016/j.jmb.2021.167200 (PMC8505758; doi:10.1016/j.jmb.2021.167200)
Supplement: Supplementary data 1 [file mmc1.docx]

**Activity of lymphostatin, a lymphocyte inhibitory**

**virulence factor of pathogenic *Escherichia coli*,**

**is dependent on a cysteine protease motif**

Andrew G. Bease^a, #^, Elizabeth A. Blackburn^b, #, $^, Cosmin Chintoan-Uta^a^, Shaun Webb^c^, Robin L. Cassady-Cain^a^ and Mark P. Stevens^a^.

^a^The Roslin Institute and Royal (Dick) School of Veterinary Studies, University of Edinburgh, Midlothian, EH25 9RG, United Kingdom.

^b^Edinburgh Protein Production Facility , University of Edinburgh, Michael Swann Building, King’s Buildings, Edinburgh, EH9 3BF, United Kingdom. (+44) 0131 6507048.

^c^Bioinformatics Core Facility , Institute of Cell Biology, University of Edinburgh, Michael Swann Building, King’s Buildings, Edinburgh, EH9 3BF, United Kingdom.

^#^These authors contributed equally.

^$^Correspondence to: e.a.blackburn@.ed.ac.uk.

**Modelling the cysteine protease domain (CPD) of lymphostatin (LifA)**

To better define the boundaries of the putative cysteine protease domain and then model the fold of the CPD of LifA, we surveyed a large database of deposited sequences against the sequence of LifA so as to not restrict our search to those of the YopT-like C58 family of cysteine proteases that have already been identified and deposited in the MEROPS database of proteolytic enzymes. We carried our five iterations of Position-Specific Iterative Basic Local Alignment Search Tool (PSI-BLAST) [1] against the non-redundant (nr) protein sequences database (22/03/2019 dataset; http://www.ncbi.nlm.nih.gov/BLAST) using the sequence of full-length EPEC E2348/69 LifA as the query and a profile-inclusion threshold of expect (*e*)-value = 0.005. This retrieved sequences with both putative glycosyltransferase and cysteine protease domains similar to lymphostatin. Our aim was to model the fold of the CPD rather than find sequences homologous with the multi-domain full-length lymphostatin. Using the full-length lymphostatin introduced compositional bias and reduced the number of CPD hits. We addressed this problem by searching with a reduced lymphostatin sequence that spans the putative CPD (S1200 – Q2200). These residues were decided upon considering our previously published limited proteolysis data [2]. We employed the heuristic that a hit with an *e*-value < 0.005 was likely to be a homologous domain. This search significantly increased the number of hits and retrieved sequences already identified as belonging to the C58 family of cysteine proteases .We aligned the retrieved sequences with Psi-Coffee [3] and combined these data with a prediction of secondary structure using the program Psi-Pred [4]. Examining these data together allowed us to propose a CPD encompassing the residues W1442 – Q1619. To test our methodology, we then searched with the reduced sequence W1442 – Q1619 and gained essentially the same results as using the more arbitrary extended sequence S1200 – Q2200. Phylogenetic analysis was performed on the sequences retrieved in Psi-Blast using the R-project and the Ape, Seqinr and MSA environments [5-8]. An unrooted tree was drawn with Interactive Tree of Life ( https://itol.embl.de; v6.3) [9]. We observed that the CPD of lymphostatin and LifA-like proteins, many of which function as bacterial virulence factors, represent a well-defined clade. (blue in phylogenetic tree shown in the body of the paper, Figure 2). The majority of MEROPS subfamily C58A, YopT-like CPDs form a clade that includes the representative member of C58A, the cysteine protease YopT from *Y. pestis*, C58.001. The representative member of C58.002, AvrPphB from *P. syringae*, is in a clade rich in bacterial plant pathogens. PaTox and the “makes caterpillars floppy” toxins Mcf1 and Mcf2 [reviewed in 10] form a clade (green in Figure 2). Figure S1 shows the alignment of retrieved sequences. Table S1 gives database accession numbers and catalytic residue information for proteins included in phylogenetic analysis. Sequence alignments are shown in Figures S1 and S2.

Examining the sequence alignment revealed that the highest sequence conservation is seen in residues close to the catalytic triad of each CPD (red text, Figure S1). These residues represent the core of the papain-like fold and define the active site. PaTox is shown in Figure S3B. The structure of two protein C58 CPD structures have been solved at atomic resolution; AvrPphB from *P. syringae* (PDB ID: **1UKF**) and PaTox from *Photorhabdus asymbiotica* (PDB ID: **6HV6**); shown in bold. LifA is most similar to PaTox, a protein with a papain-like cysteine protease fold. To further examine homology between the CPD of lymphostatin and PaTox, we used the crystal structure of the PaTox CPD as a structural input to the program T-Coffee Expresso to align the group of sequences that show the highest sequence conservation with lymphostatin (blue branch, Figure 2 main paper), Figure S2. The lymphostatin CPD shows well-conserved secondary structure propensity and amino acid conservation in residues that facilitate the papain-like fold of PaTox. From this evidence, we modelled the CPD of lymphostatin based on PaTox using the Phyre2 server [11]. To interrogate our homology model of the lymphostatin CPD we ran the docking program ArDock [12]. The residue interaction propensity was used to colour code the domain surface (Figure S2B).

**Table S1. Database accession numbers and catalytic residue information for proteins included in phylogenetic analysis (Figure 2 main paper) and sequence alignments (Figure S1 and S2 supplemental information).** * indicates a partial sequence.

| **Abbreviation** | ***Species*** | **Protein** | **Active site residues** | **NCBI/GenBank/UNIPROT** |
| --- | --- | --- | --- | --- |
| *E.coli_LifA* | *Escherichia coli O127:H6* | Lymphostatin /LifA | C1480, H1581, D1596 | **WP_001239084.1** |
| *E.albertii_LifA* | *Escherichia albertii* | Lymphostatin /LifA | C1478, H1579, D1594 | **WP_000701120.1** |
| *C.rodentium_LifA* | *Citrobacter rodentium* | LifA/Efa1-related | C1615, H1716, D1731 | **WP_012907284.1** |
| *Pr.alcalifaciens_LifA* | *Providencia alcalifaciens* | LifA/Efa1-related | C1292, H1393, D1408 | **WP_051420264.1** |
| *K.pneumoniae_YopT-type* | *Klebsiella pneumoniae* | YopT-type protease | C1218, H1318, D1333 | **WP_134921289.1** |
| *Ch.ibidis_LifA* | *Chlamydia ibidis* | LifA/Efa1-related | C1352, H1453, D1469 | **WP_021119564.1** |
| *Ch.psittaci_LifA* | *Chlamydia psittaci* | LifA/Efa1-related | C512, H613, D629* | **EPJ32470.1*** |
| *Ch.buteonis_LifA* | *Chlamydia buteonis* | LifA/Efa1-related | C1485, H1586, D1602 | **WP_131744402.1** |
| *Ch.felis_LifA* | *Chlamydia felis* | LifA/Efa1-related | C1499, H1601, D1616 | **WP_011457994.1** |
| *Ch.suis_LifA* | *Chlamydia suis* | LifA/Efa1-related | C1462, H1563, D1578 | **WP_080141411.1** |
| *Ch.pecorum_LifA_gene1* | *Chlamydia pecorum* | LifA/Efa1-related | C1584, H1685, D1700 | **WP_021757348.1** |
| *Ch.muridarum_LifA_gene1* | *Chlamydia muridarum* | LifA/Efa1-related | C1478, H1579, D1594 | **WP_010904337.1** |
| *Ch.pecorum_LifA_gene2* | *Chlamydia pecorum* | LifA/Efa1-related | C1646, H1747, D1762 | **WP_021757351.1** |
| *Ch.muridarum_LifA_gene2* | *Chlamydia muridarum* | LifA/Efa1-related | C1565, H1666, D1681 | **WP_010230448.1** |
| *Ch.trachomatis_LifA* | *Chlamydia trachomatis* | LifA/Efa1-related | C284, H385, D400 | **APD39866.1** |
| *E.coli_LifA-like_O127 H6* | *Escherichia coli O127:H6* | LifA/Efa1-related | C1710, H1814, D1829 | **CAS08627.1** |
| *E.coli_LifA-like_O111 H-B171-8* | *Escherichia coli O111:H-B171-8* | LifA/Efa1-related | C1796, H1900, D1915 | **BAG66781.1** |
| *G.hollisae_ToxB* | *Grimontia (vibrio) hollisae* | Toxin B | C1797, H1901, D1916 | **WP_115660322.1** |
| *E.coli_ToxB* | *Escherichia coli* | LifA/Efa1-related | C1437, H1539, D1554 | **UNIPROT:C1J8E9** |
| *S.enterica_LifA* | *Salmonella enterica* | LifA/Efa1-related | C3032, H3134, D3149 | **WP_079805579.1** |
| *Ph.asymbiotica_PaTOX* | *Photorhabdus asymbiotica* | PaTox | C1865, H1955, D1975 | **UNIPROT:C7BKP9** |
| *V.vulnificus_McfVv* | *Vibrio vulnificus* | holotoxin RtxA | C3351, H3463, D3482 | **WP_011081430.1** |
| *Ph.luminescens_Mcf1* | *Photorhabdus luminescens* | Mcf1 | C1397, H1486, D1505 | **UNIPROT:Q8KT65** |
| *Ph.luminescens_Mcf2* | *Photorhabdus luminescens* | Mcf2 | C791, H881, D900 | **UNIPROT:Q6SY96** |
| *Ca.hamiltonella_YopT* | *Candidatus Hamiltonella defensa* | YopT-type peptidase | C57, H176, D195 | **NCBI:WP_015873572.1** |
| *Pseudomonas.sp_peptidase* | *Pseudomonas sp. GM21* | YopT-type peptidase | C43, H148, D163 | **GENBANK:WP_007944484.1** |
| *Ps.syringae_AvrPphB* | *Pseudomonas syringae* | AvrPphB | C98, H212, D227 | **UNIPROT:Q52430** |
| *P.syringae_peptidase* | *Pseudomonas syringae* | YopT-type peptidase | C51, H161, D176 | **NCBI:WP_011282458.1** |
| *Ac.citrulli_NopT* | *Acidovorax citrulli* | NopT | C22, H132, D147 | **GENBANK:ABM30699** |
| *B.japonicum_NopT_1* | *Bradyrhizobium japonicum* | NopT | C100, H213, D228 | **UNIPROT:Q89T99** |
| *Si.fredii_NopT* | *Sinorhizobium fredii* | NopT | C93, H205, D220 | **UNIPROT:P55730** |
| *B.japonicum_NopT_2* | *Bradyrhizobium japonicum* | NopT | C109, H223, D238 | **UNIPROT:Q9AMW4** |
| *Ps.savastanoi_ORF4* | *Pseudomonas savastanoi* | ORF4 | C156, H265, D280 | **UNIPROT:Q9RBW5** |
| *Ps.syringae_ORF4* | *Pseudomonas syringae* | ORF4 | C156, H265, D280 | **GENBANK:AAD47206** |
| *R.solanacearum_RipT* | *Ralstonia solanacearum* | RipT | S122, H245, E262 | **UNIPROT:Q8XUH6** |
| *R.solanacearum_peptidase* | *Ralstonia solanacearum* | YopT-type peptidase | C31, H152, D169 | **GENBANK:CBJ35895.1** |
| *Co.fungivorans_peptidase* | *Collimonas fungivorans* | YopT-type peptidase | C175, H297, D313 | **GENBANK:AEK63537.1** |
| *Azospirillum.sp_peptidase* | *Azospirillum sp. B510* | YopT-type peptidase | C61, H179, D201 | **GENBANK:BAI73218** |
| *Ps.avellanae_HopN1* | *Pseudomonas avellanae* | HopN1 | C172, H283, D299 | **NCBI:WP_005615685** |
| *Pseudomonas.sp_HopN1* | *Pseudomonas sp. GM50* | HopN1 | C172, H283, D299 | **NCBI:WP_008011097.1** |
| *Ps.syringae_HopN1* | *Pseudomonas syringae* | HopN1 | C172, H283, D299 | **UNIPROT:Q9JP32** |
| *X.bovienii_peptidase* | *Xenorhabdus bovienii* | YopT-type peptidase | C36, H119, D134 | **GENBANK:CBJ82189.1** |
| *M.variabilis_peptidase* | *Microbulbifer variabilis* | YopT-type peptidase | C38, H178, D199 | **GENBANK:WP_020413014** |
| *T.oleivorans_peptidase* | *Thalassolituus oleivorans* | YopT-type peptidase | C44, H153, D170, | **NCBI:WP_015487003.1** |
| *Sc.paludicola_peptidase* | *Schlesneria paludicola* | YopT-type peptidase | C48, H195, D209 | **NCBI:WP_029247385.1** |
| *Ha.chejuensis_peptidase* | *Hahella chejuensis* | YopT-type peptidase | C57, H199, D213 | **GENBANK:ABC27630** |
| *Ha.ganghwensis_peptidase* | *Hahella ganghwensis* | YopT-type peptidase | C49, H188, D202 | **GENBANK:WP_020406726.1** |
| *Hae.ducreyi_LspA2* | *Haemophilus ducreyi* | LspA2 | C2966, H3090, D3105 | **UNIPROT:Q9ZHL0** |
| *Hi.somni_IbpA* | *Histophilus somni* | IbpA | C3910, H4033, D4048 | **NCBI:WP_012340627.1** |
| *Pa.dagmatis_YopT* | *Pasteurella dagmatis* | YopT-type peptidase | C132, H255, D270 | **NCBI:WP_005764706.1** |
| *Pa.multocida_PfhB2* | *Pasteurella multocida* | PfhB2 | C3733, H3856, D3871 | **UNIPROT:Q9CPH9** |
| *Pa.multocida_peptidase_2* | *Pasteurella multocida* | YopT-type peptidase | C2432, H2555, D2570 | **GENBANK:WP_005755837** |
| *Pa.multocida_peptidase_1* | *Pasteurella multocida* | YopT-type peptidase | C368, H491, D506 | **GENBANK: EPE68154.1** |
| *Pa.multocida_YopT* | *Pasteurella multocida* | YopT-type peptidase | C354, H477, D492 | **NCBI:WP_016534357.1** |
| *V.harveyi_YopT* | *Vibrio harveyi* | YopT-type peptidase | C144, H264, D279 | **NCBI:ZP_01986023.1** |
| *V.campbellii_YopT* | *Vibrio campbellii* | YopT-type peptidase | C144, H264, D279 | **NCBI:WP_005431100.1** |
| *Y.enterocolitica_YopT* | *Yersinia enterocolitica* | YopT-type peptidase | C139, H258, D274 | **UNIPROT:P27475** |
| *Y.pseudotuberculosis__YopT* | *Yersinia pseudotuberculosis* | YopT-type peptidase | C139, H258, D274 | **UNIPROT:Q93RN4** |
| *Y.pestis_YopT* | *Yersinia pestis* | YopT-type peptidase | C139, H258, D274 | **UNIPROT:O68703** |
| *Ae.diversa_YopT* | *Aeromonas diversa* | YopT-type peptidase | C139, H256, D272 | **NCBI:WP_005351265** |
| *Ph.luminescens_2_YopT* | *Photorhabdus luminescens* | YopT-type peptidase | C141, H259, D275 | **UNIPROT:Q7N4D9** |
| *Ph.asymbiotica_YopT* | *Photorhabdus asymbiotica* | YopT-type peptidase | C141, H260, D276 | **NCBI:WP_015834232.1** |
| *Ph.luminescens_LopT* | *Photorhabdus luminescens* | LopT | C136, H247, D263 | **GENBANK:AAO18078** |
| *Ph.luminescens_YopT* | *Photorhabdus luminescens* | YopT-type peptidase | C136, H263, D279 | **UNIPROT:Q7N0U1** |
| *P.temperata_YopT* | *Photorhabdus temperata* | YopT-type peptidase | C136, H263, D279 | **GENBANK:YP_001784809** |
| *E.coli EspL* | *Escherichia coli O127:H6* | EspL | C47, H131, D153 | **UNIPROT:B7UI20** |
| *S.flexneri_OspD3* | *Shigella flexneri* | OspD3 | C64, H148, D171 | **UNIPROT:Q6XVZ7** |

Core index                                                                                                                       *

*1470 1480*

*....|....|....|....|....|....|....|....|....|....|....|....|....|....|....|....|....|....|....|....|....|....|....|....|....|....|....|....|*

***E.coli_LifA***  W--------P--E-------F-Y----RHH----------AQR-------------------WFEMAKGY---GSQNIDFHP-Q---SLL--------VTQEGRCMGLALLYLQTEDTA------H--YSI-LQENLMTV

***E.albertii_LifA***  W--------P--E-------F-Y----RQH----------AQR-------------------WFEMAKGY---GSQNIDFHP-Q---SLL--------ISQEGRCMGMALLYLQTENTA------H--YRI-LQENLMTV

***Pr.alcalifaciens_LifA***  W--------S--E-------F-Y----RHH----------AHS-------------------WFSLAKGH---AADTVDFHP-Q---SLL--------ISPEGRCTGLAWLYLQAEDII------H--YAV-LQENLMTV

***C.rodentium_LifA***  W--------P--E-------F-Y----RDH----------AQL-------------------WFSLARGH---AADTTDFHP-Q---SLL--------ISQEGRCMGLAWLYLQAEDSI------H--YAV-LQENLMTV

***K.pneumoniae_YopT-type***  W--------P--D-------F-Y----GSH----------ARL-------------------WNEVAATF---RATKTEFHP-Q---SLI--------VSNEGRCMGLTLLYMNAGTTS------E--YDI-VTQNLLTA

***Ch.ibidis_LifA***  W--------S--E-------F-F----NVH----------SER-------------------WSDLANRL---GSKSIDIHP-Q---TFL--------YRVEGRCVGLSMLYLLAKDVT------S--YSL-IQDNLMTV

***Ch.buteonis_LifA***  W--------Q--E-------F-F----NTH----------AEG-------------------WSDLANRL---GGKSIDIHP-Q---TFL--------YKVEGRCIGISMLYLLAKDTA------S--YGL-IQDNLMVI

***Ch.psittaci_LifA***  W--------P--E-------F-F----NTH----------AES-------------------WSDLANRL---GGKSIDVHP-Q---TFL--------YKVEGRCIGVSMLYLLAKDTT------S--YSL-IQDNLTVI

***Ch.felis_LifA***  W--------K--E-------F-Y----ESH----------VAL-------------------WDVVAARL---GSEKTQIHP-Q---TFL--------YELEGRCMGLSMLYMSATDVV------T--YAT-LVENLMTA

***Ch.suis_LifA***  W--------P--E-------F-Y----DNH----------ADQ-------------------WNDLANRF---GAEHLNVHP-Q---TFI--------YEAEGRCMGLALLYMLAEDSI------S--YRL-LQQNVMTA

***Ch.pecorum_LifA_gene1***  W--------P--E-------F-Y----HSL----------STL-------------------WSDLARHY---GADVLSAHP-Q---SFL--------YEIEGRCMGLSMLYMSAESAA------S--YRL-LQENLETV

***Ch.muridarum_LifA_gene1***  W--------P--E-------F-Y----NRY----------ANA-------------------WSDLATHY---GAEILEAHP-Q---SFL--------YEVEGRCMGLSLLYMSIEDEG------G--YRT-LQGNLDTV

***Ch.pecorum_LifA_gene2***  W--------P--E-------F-S----SKF----------SGI-------------------FGDLAFRL---GAKTLELHP-Q---TFL--------YRIEGRCMGLSYLFLNAQDKM------S--YST-LQHNLVTV

***Ch.muridarum_LifA_gene2***  W--------T--K-------F-Y----ERY----------SGI-------------------WGDLAFRL---GAESLRTHP-Q---TFI--------YDTEGRCMGLSYLFLAAENIA------A--YGI-LQDNLSTL

***Ch.trachomatis_LifA***  W--------A--D-------F-Y----ERY----------AGI-------------------WGDLAFRL---GAETLSTHP-Q---SFI--------FDAEGRCMGLSYLCMTADSQA------A--YST-LQDNLSTV

***S.enterica_LifA***  W--------E--D-------F-Y----TRF----------AGL-------------------WDNAFRSV---GGTGIAFHP-Q---SLL--------FEQEGKCMGLSLLYVETAGQPA-----H--YQI-LQDNLMKA

***E.coli_ToxB***  W--------N--D-------F-Y----NTH----------ASI-------------------WDTIARQH---KSTNIEFHP-Q---SLL--------FDRDGKCLGLSLLYLDTGGGS------R--YQK-LRHNIETA

***G.hollisae_ToxB***  W--------G--D-------F-Y----GRN----------AKL-------------------WQEAVRKF---EGNNVKYHP-Q---MLL--------TPEEGRCMGLAELYLLANSEE------H--YKT-LQENLDLA

***E.coli_LifA-like_O111***  W--------R--S-------F-Y----GHN----------ARL-------------------WHETVIKY---SGSEPRYHP-Q---MLL--------SPNEGRCIGLSELYILADTKE------K--YNT-LQENLDLI

***E.coli_LifA-like_O127***  W--------R--N-------F-Y----GHN----------ARL-------------------WHDAVIKY---SGSEPRYHP-Q---MLL--------SPNEGRCIGLSELYILADTKE------K--YNT-LQENLDLI

***Ph.asymbiotica_PaTOX***  K--------D--INAF-----------QTA----------KDA-----------------QSWKKSANK-----ANKVVLTP-Q--NLYL--------KGKPSECLPESVLMGWALQSS------Q--DAK-LSKMLMGI

***V.vulnificus_McfVv***  L--------K--V-------T-F----QN--K--------SEKYNRLFREIASAGVVDAKAT-----EQLAPQL--------ML-LNLSN--------DGFGGRCDPLSKLVLVAKQLENDGQVGV--ARQ-LLEKMY--

***Ph.luminescens_Mcf1***  K--------V-----------------LR--Q--------TAVFSEDFHDAGS-------VF-----DRLVPQD--------FY-LSLVG--------DRSGGRCYPLVRAMTVALASGG---EAG--INS-LVQKLF--

***Ph.luminescens_Mcf2***  K--------V-----------------LK--W--------TAVYSEDFHRAGS-------SF-----DRLAPQD--------FY-LSLVG--------DKSGGRCYPLVRAMAVALANNG---ETG--INS-LVEKLF--

***Azospirillum.sp_peptidase*** -----------------------------------------------------------------------------------------H--------EATKGICFGLCLEWIKRHRANKG----E--T---PQTRI---

***Co.fungivorans_peptidase***  S--------L--K-------T-ALLARTN--Q--------GAA-----------------FL-----THF---SD------------EEM--------SRWEGACNGQCHIWMRLREASPL----A--A---ATDRL---

***R.solanacearum_peptidase***  M--------A--Q-------C-A----VA--R--------TRQ---------G-------LA-----FRARF-------------GEESA--------QHYSGSCVGLSAVWIRLHEAA------P--ATY-AVNRVN--

***R.solanacearum_RipT***  A--------R-------------------------------------------------------------F-------------GAESA--------QHYSGSCVGLSAVWIRLHEAA------P--ATH-AVNRVN--

***P.syringae_peptidase***  G--------C--S-------S-ST---AS--R--------IKE-----------------IP-----FKQ---A-------D--E-LARV--------GDQRAACVVLTAAWLDRVHHH------SQPAEA-RIDHMR--

***Ps.syringae_AvrPphB***  D--------K--G-------C-A----SS--S--------GVS-----------------LE-----DDS---H-TQVSLSD-FSVASRD--------VNHNNICAGLSTEWLVMSSDG------D--AES-RMDHLD--

***Ps.syringae_ORF4***  A--------D--I-------DAV----FN----------------------------------------YRI-------------AALNN--------ANASQSCMGLAIQWLRLRDEE------E--ASY-RMEALD--

***Ps.savastanoi_ORF4***  -----------------------------------------------------------------------M-------------AALNN--------ANASQSCMGLAIQWLRLRDEE------E--ASY-RMEALD--

***Ac.citrulli_NopT***  M--------S--T-------S-V----FA----------------------------------------YQT-------------AELEQ--------ANVEGICVGLVTEWLRRPNQS------P--SGR-M-AALA--

***B.japonicum_NopT_1***  P--------S--T-------S-S----PE--S--------PA----------T-------SL-----FEYRT-------------ADLRD--------ANVDGICVGLTAEWFRNLSNS------P--STR-M-SALT--

***Si.fredii_NopT***  P--------S--T-------S-S----PA--R--------PS----------T-------SL-----FRYRT-------------AELAQ--------ANADGICVGLTAEWLRNLNSH------P--SIR-M-EALV--

***B.japonicum_NopT_2***  P--------V--R-------P-L----FD----------------------------------------YRT-------------AELPQ--------ANVSGICVGLAAEWLLDLPSS------A--SSR-M-GVLL--

***Pseudomonas.sp_peptidase***  M--------L--N-------A-I----HNSEK--------FG----------S-------IQ-----VDYKQ-SEITKNVYEVY-GVRTN--------ATDSGVCMAMSAKYLLKNSQN------Q--DFF---SWLA--

***Ca.hamiltonella_YopT***  I--------D--E-------T-V----KK--Y--------GGT-----------------VV-----KHY---NQNTGKLAS-I-IKLSD--------STALGICAGYSTKQLIALSEG------K--TID---RDMR--

***X.bovienii_peptidase***  M--------G--IYLKMSLLD-A----DEF----------EGN-----------------WVFRFNQ---------TLN-FP-L--KFSS--------TMQNGVCSSLVMAWVKMHKLG------K--SHL-FLGNIRLP

***T.oleivorans_peptidase***  V--------Q--Q-------L-A----DN--F--------QGH-----------------CT-----WDF---SQGFP-----L-HFIQA--------PTGNGICMGLSCHWIKYHALD------D--S---LVTHLG--

***M.variabilis_peptidase***  M--------GVDR-------I-A----RR--N--------NGV-----------------KT-----WGF---SQSSPRAYI-H-Y--FR--------FGKFGICASLAAHWIKTNALDD-----K--S---LPDKL---

***Sc.paludicola_pepidase***  I--------Q--K-------S-A----EE--Y--------KGH-----------------CT-----HTF---SQTLEPVSS-M-IGKSK--------KTKDGICQALSEMWIVFHAHD------G--S---IWNWLC--

***Ha.ganghwensis_peptidase***  V--------Q--Q-------S-A----VA--A--------GGF-----------------CT-----WPF---SQVRDPVKS-L-ICSHT--------DTSGGICEMLAAKWLESHANNGS----I-------VNWI---

***Ha.chejuensis_peptidase***  V--------Q--N-------S-A----TA--A--------GGH-----------------CT-----WRF---SQVKNPVRS-L-ILTNS--------DTSGGICEMLAAKWLEVHANAEK----Y-------ARSGE--

***Hae.ducreyi_LsAp2***  V--------R--S-------S-V----EE--Y--------GGE-----------------VT-----FKY---AQSKGEVYN-E-IVKHA--------ETQNGVCEATCSHWIAKKVND------E--N---IWTDL---

***Hi.somni_IbpA***  V--------R--S-------S-V----EE--F--------GGE-----------------VS-----FKF---AQSKGEVYK-E-IVKHI--------ETQNGVCESTCAHWIAKNVNPTD----E--N---FFNTL---

***Pa.dagmatis_YopT***  V--------R--S-------S-V----EQ--Y--------GGE-----------------VT-----FKY---AQSKGEVYK-E-IVKHA--------ETEHGVCESTCAHWIANKVSNQG----E--D---FWNTL---

***Pa.multocida_peptidase_2***  V--------R--S-------S-V----EE--F--------GGE-----------------VS-----FKY---AQSKGEVYN-E-IVKHV--------DTQHGVCESTCAHWIANKVSSQG----E--D---FWNTM---

***Pa.multocida_PfhB2***  V--------R--A-------S-V----AE--Y--------GGE-----------------VS-----FKY---AQSKGEVYK-E-IVKHV--------DTQHGVCESTCAHWIANKVSSQG----E--D---FWNTM---

***Pa.multocida_YopT***  V--------R--A-------S-V----EE--Y--------GGE-----------------VS-----FKY---AQSKGEVYK-E-IVKHI--------ETQNGVCESTCAHWIANKVTSQS----E--D---FWNTM---

***Pa.multocida***  V--------R--A-------S-V----EE--Y--------GGE-----------------VS-----FKY---AQSKGEVYK-E-IVKHI--------ETQNGVCESTCAHWIVNKVTSQS----E--D---FWNTM---

***Y.enterocolitica_YopT***  V--------R--E-------S-V----AN--Y--------GGN-----------------IN-----FKF---AQTKGAFLH-Q-IIKHS--------DTASGVCEALCAHWIWSHAQG------Q--S---LFDQL---

***Y.pestis_YopT***  V--------R--E-------S-V----AN--Y--------GGN-----------------IN-----FKF---AQTKGAFLH-K-IIKHS--------DTASGVCEALCAHWIRSHAQG------Q--S---LFDQL---

***Y.pseudotuberculosis__Yop*** V--------R--E-------S-V----AN--Y--------GGN-----------------IN-----FKF---AQTKGAFLH-K-IIKHS--------DTASGVCEALCAHWIRSHAQG------Q--S---LFDQL---

***Ph.asymbiotica_YopT***  V--------R--N-------S-A----SK--Y--------GGQ-----------------VT-----FKF---AQTKGTFLD-Q-IMKHK--------DTSGGVCESISAHWISAHAKG------E--S---IFDQL---

***P.temperata_YopT***  V--------R--D-------S-A----GK--H--------GGE-----------------VT-----FKF---AQTKGAFLD-Q-IMKHK--------DTAGGVCESISAHWISAHAKG------E--S---VFDQL---

***Ph.luminescens_3_YopT***  V--------R--N-------S-A----SK--H--------GGQ-----------------VT-----FKF---AQTKGTFLD-Q-IMKHK--------DTSGGVCESISAHWISAHAKG------E--S---IFDQL---

***Ph.luminescens_2_YopT***  V--------R--N-------S-A----SK--H--------GGE-----------------IT-----FKF---AQTKGTFLD-Q-IMKHS--------DTAGGVCESISAHWISAHAKG------E--S---VFDQL---

***Ph.luminescens_1_YopT***  V--------R--D-------S-A----SK--H--------GGE-----------------VT-----FKF---AQTKGAFLD-Q-IMKHQ--------DTSGGVCESISAHWISAHAKG------E--S---VFNQL---

***Ae.diversa_YopT***  V--------R--D-------S-V----PQ--H--------GGS-----------------VT-----FKF---AQTKGAFLG-E-IMKHG--------DTSGGVCESISAHWISSHAKG------E--S---LFNQL---

***V.campbellii_YopT***  L--------R--N-------S-A----AK--H--------NGE-----------------VT-----MKV---SQVTGSIKN-A-ILKDT--------NTSGGCCEALSAHWMKARAEG------S--N---LGEQL---

***V.harveyi_YopT***  L--------R--N-------S-A----AK--H--------NGE-----------------VT-----MQV---SQVTGSIKN-A-ILKDT--------NTSGGCCEALSAHWMKARAEG------S--N---LGEQL---

***Pseudomonas.sp_HopN1***  GGTANIEAKR--Q-------I-A----EE--H--------GCH-----------------LA-----SPF---HQSKFLFEK-T-VDDSEFAADYSRAGGGGHGCFGLSVNWCQSRAKG------Q--SDEVFFDKLA--

***Ps.avellanae_HopN1***  GATANIEAKR--K-------I-A----QE--H--------GCQ-----------------LV-----HPF---HQSKFLFEK-T-IDDRAFAADYGRAGGNGHACLGLSVNWCQSRAKG------Q--SDEAFFYKLE--

***Ps.syringae_HopN1***  GATANIEAKR--K-------I-A----QE--H--------GCQ-----------------LV-----HPF---HQSKFLFEK-T-IDDRAFAADYGRAGGDGHACLGLSVNWCQSRAKG------Q--SDEAFFHKLE--

***E.coli_EspL***  Y--------I--S-------K-N----NP--------------------------------------PYL---SKKRDASIN-LNGKVSD-------CNGEIIWCRHIASYWSEFFCSN------S--GKI---DYET--

***S.flexneri_OspD3***  M--------I--N-------K-D----NV--SVETIQSLLHSK-----------------QL-----PYF---SDKRSFLLN-LNCQVTD-------HSGKLIVCRHLASYWIAQFNKS------S--GHV---DYHH--

Core index

*....|....|....|....|....|....|....|....|....|....|....|....|....|....|....|....|....|....|....|....|....|....|....|....|....|....|....|....|*

***E.coli_LifA***  SALHQTSNRDKLP----LS--K---D---D---N---SLMTRTYSL------------IEM--------LQ--------------------YQGN-------------------KYI--TNES-LLH--K---T------

***E.albertii_LifA***  SALHQTYKRDKLP----LT--E---D---D---K---AIMARAYRL------------IEM--------LQ--------------------YQGN-------------------KHI--ADKS-LLQ--K---A------

***Pr.alcalifaciens_LifA***  SALHQTRERDNLL----LS--E---A---D---N---ALLDKALQL------------INQ--------LQ--------------------EQGN-------------------QHI--QSNT-LLT--M---V------

***C.rodentium_LifA***  SALRQTRERSHLP----LT--E---A---D---N---SLLDNALQL------------IDK--------LQ--------------------VQGN-------------------QYI--QSNT-FFN--K---A------

***K.pneumoniae_YopT-type***  GGLLQTRDSMHLP----LT--L---Q---D---N---KFLNDTISM------------IDR--------LQ--------------------LQGN-------------------TLI--SEYS-NQQ--R---M------

***Ch.ibidis_LifA***  SSLYQEKEIKGLS----LT--E---T---D---Q---KLLDRSTSL------------IDW--------LQ--------------------FQGN-------------------KYL--KSSD-AFS--S---I------

***Ch.buteonis_LifA***  SSLYQEKESEGLP----LT--K---T---D---R---DLLDRITTL------------IDW--------LQ--------------------FQGN-------------------KYL--KSPD-VFS--S---I------

***Ch.psittaci_LifA***  SSLYQEKERERLP----LT--K---T---D---Q---DLLDRSTTL------------IDW--------LQ--------------------FQGN-------------------KYL--KSPE-VFS--R---I------

***Ch.felis_LifA***  GALFQTKEIERLP----LT--W---S---D---D---RFLSKTLNL------------VDW--------LQ--------------------YQGN-------------------KNL--KASG-ILT--P---H------

***Ch.suis_LifA***  SALFNEQKRLGIP----LT--K---E---D---Q---KFLDKVLSL------------IEW--------LQ--------------------FRGN-------------------QQL--QTEG-FFR--T---L------

***Ch.pecorum_LifA_gene1***  SALFQTKEREHLP----LT--K---S---D---Q---DLLNRSLSL------------INW--------LQ--------------------YQGN-------------------RFL--LEKK-ILT--E---K------

***Ch.muridarum_LifA_gene1***  SALYQQKERDHLP----LS--S---K---D---Q---SLLSRDLSL------------INW--------LQ--------------------YQGN-------------------KVL--LENR-GFN--H---A------

***Ch.pecorum_LifA_gene2***  SSLFQTKERDKLP----LS--Q---Q---D---N---KFLKRSLDL------------IEW--------LQ--------------------HRGN-------------------REL--QIGG-VLS--S---L------

***Ch.muridarum_LifA_gene2***  SALFLEREREKLP----LS--R---E---D---N---RFLDRGLAL------------IEW--------LQ--------------------HRGN-------------------SEL--QAGG-IFS--T---V------

***Ch.trachomatis_LifA***  SALFLMREKEQLP----LS--K---Q---D---D---KFLNRGLDL------------IEW--------LQ--------------------YLGN-------------------ENL--QVRG-LAS--S---L------

***S.enterica_LifA***  GALFQTRHRDGLP----LS--D---H---D---D---AFLQRTLEI------------VEA--------AQ--------------------QRGN-------------------EKL--SGSR-LSS--L---N------

***E.coli_ToxB***  SALYQTKYNDNIK----LS--N---S---D---D---FFLRKTQRI------------ITM--------SN--------------------ELGN-------------------NRL--KNSQ-LEI--L---E------

***G.hollisae_ToxB***  SALYQESQIDQYQ----LS--E---G---D---K---HLLDSMLNQ------------VEH--------AQ--------------------QHGN-------------------NKL--LSSQ-DIE--KIRLS------

***E.coli_LifA-like_O111***  SSLYQQDISDNAH----LS--E---S---D---H---RLLKNTVSQ------------IEY--------YQ--------------------QHGN-------------------NKL--LQSR-ELE--RIRLT------

***E.coli_LifA-like_O127***  SSLYQQDISDNAH----LS--E---S---D---H---RLLKNTVSQ------------IEY--------YQ--------------------QHGN-------------------NKL--LQSG-ELE--RIRLT------

***Ph.asymbiotica_PaTOX***  YSS---NDITSN-----------------------------PLYKS------------LKE--------LH--------------------ANGN-------------------ASK--FNAS-ATS--I---S------

***V.vulnificus_McfVv***  -----------------SA--A---A---V------LSNPTL-YSDSEKANASKLLSSLAA--------I-------H-------------AKN-------P------------MHD------------------TSM-K

***Ph.luminescens_Mcf1***  -----------------FA--S---A---D------PQAGSS-----TL-----LRNSLIK--------L-------H-------------SNV-------E-------------AV-----------------------

***Ph.luminescens_Mcf2***  -----------------LA--A---A---D------PHASSS-----TL-----LTNSLIR--------L-------H-------------SNN-------V------------EAV-----------------------

***Azospirillum.sp_peptidase*** ------------A----YI--D---Q---D---S---TILH---AS------------IKQ---------------RL--------------------------YRAEL-HFDLD-L--ASEQLGAR-N-QAMGQAGFKV

***Co.fungivorans_peptidase***  ------------N----AL--A---S---F---E---GAVH---AN------------IHQ---------------SA--------------------------YSDNR-ADDF--S--IGRL-LGI-D-AWSSKPGTHD

***R.solanacearum_peptidase***  -----------------TA--G---S---F-----DGMAHAEVYQR-----------AYEA--------N-------R-------------TD--------------------------------------MLQGRASKH

***R.solanacearum_RipT***  -----------------TA--G---S---F-----DGMAHAKVYQR-----------AYEA--------N-------Q-------------SD--------------------------------------MLQGRASKR

***P.syringae_peptidase***  -----------------HR--ATL--------------------EQ------------VAE--------R-------Q-------------QTYRN-HEINN------------PRTPYEIL------FSPTFRDYSLRL

***Ps.syringae_AvrPphB***  -----------------YN--GEGQ-------------------SR------------GSE--------R-------H-------------QVYNDALRAAL------------SNDDEAPFF-TAS-T-AVIEDAGFSL

***Ps.syringae_ORF4***  -----------------LD--H--------------ASDIQNQYEN-----------AAGS--------V-------S-------------GSR-------E------------QRE--AGRI-SAR-K-TLLRSQDLQP

***Ps.savastanoi_ORF4***  -----------------LD--H--------------ASDIQNQYEN-----------AAGS--------V-------S-------------GSR-------E------------QRE--AGRI-SAR-K-TLLRSQDLQP

***Ac.citrulli_NopT***  -----------------RD--T---P---S---HAQAALRQQKYQQ-----------DKDA--------L-------R-------------AQG-------M------------GAA--DADM-RAQ-N-GVLREAGLRP

***B.japonicum_NopT_1***  -----------------PG--S---Q---T---HASAAERQQQYQR-----------LKDQ--------L-------R-------------SRG-------A------------GSS--QADL-QAQ-N-TILEEAGLEP

***Si.fredii_NopT***  -----------------PG--S---Q---R---HASATVRQKEYEN-----------LKVH--------L-------R-------------RQG-------A------------GPS--EADF-AAQ-N-TMLQKAGLAP

***B.japonicum_NopT_2***  -----------------PG--T---E---N---HRSAARRQEQSEK-----------LKTQ--------L-------K-------------EDK-------A------------E-G--SHNF-QAK-S-TILRDAGLEP

***Pseudomonas.sp_peptidase***  -----------------DS--S---S---K------WEVVSSYLDS-----------DYSA--------L-------A-------------PIE-------V-----------QDRI--QDEL-KLH-L-NHLGTLNLKP

***Ca.hamiltonella_YopT***  -----------------VV--NKNDK---K---S---KLKISEVNK------------ICH--------L-------Q-------------AEG-------D------------EIFLKNPDF-KFD-D-QVLEKNNFSI

***X.bovienii_peptidase***  SYI--------------------------------------KIVEN------------LKA--------KS--------------------MDGL-------------------LFL--NENG-LSYRHV---S------

***T.oleivorans_peptidase***  -----------------AYRIP---NTRTF---I---PFNHNEYQR------------IS--------QW-------Q-------------NNL-------A-------------TF--PDWR-MGY-Q-NWFRNQGIGI

***M.variabilis_peptidase***  ------------G----LT--P---R---VHNAG---AFSFRTYRS------------LNI------GELIKVGRDFR-------------TWT-------Y------------GG---GRQG-LNI-E-NWLINQGLHK

***Sc.paludicola_pepidase***  -----------------PD--------------G---KVNSSALAN------------VAYNFNYGTSMF-------E-------------GKG-------S-------------AI--TDQD-QNS-D-LWFLQYGIRR

***Ha.ganghwensis_peptidase***  -----------------MD--S---S---G---T---QVDPNKIRQ------------LMQ---------------WF-------------AVG-------VSMRAVAMNERSNEGG--MDQS-LAT-E-RWLRTHGVVR

***Ha.chejuensis_peptidase***  ----------ATSLAGWIS--R---T---G---G---EIDPNKVRQ------------IMQ---------------MF-------------IIG-------STMNSGAVVGQPGIGR--EDQN-YAT-E-RWLQSKGVTR

***Hae.ducreyi_LsAp2***  ------------Y----KD--G---QKGRK---G---GLNKDAIES------------IEK--------L-------Q-------------TEF-------I------------NAGTATQQF-KLT-N-TWLEEQGVVP

***Hi.somni_IbpA***  ------------Y----EG--G---K---K---G---HLKKETIDS------------IKK--------L-------Q-------------TEF-------I------------NSGSATQQF-KLT-D-SWLQEQGVVP

***Pa.dagmatis_YopT***  ------------Y----EG--G---K---K---G---HLKQDAIDS------------IKK--------L-------Q-------------TEF-------M------------NSGSATQQF-KLT-D-SWLQEQGVVP

***Pa.multocida_peptidase_2***  ------------Y----EG--G---K---K---G---HLKQEAIDS------------IKK--------L-------Q-------------TEF-------M------------QSGSATQQF-KLT-D-NWLQEQGVVP

***Pa.multocida_PfhB2***  ------------Y----EG--G---K---K---G---HLKQEAIDS------------IKK--------L-------Q-------------TEF-------M------------QSGSATQQF-KLT-D-NWLQEQGVVP

***Pa.multocida_YopT***  ------------Y----EG--G---K---K---G---HLKQETIDS------------IKK--------L-------Q-------------TEF-------M------------NSGSATQQF-KLT-D-SWLQEQGVVP

***Pa.multocida***  ------------Y----EG--G---K---K---G---HLKQETIDS------------IKK--------L-------Q-------------TEF-------M------------NSGSATQQF-KLT-D-SWLQEQGVVP

***Y.enterocolitica_YopT***  ------------Y----VG--G---R---K---G---KFQIDTLYS------------IKQ--------L-------Q-------------IDG-------C------------KAD--VDQD-EVT-L-DWFKKKGISE

***Y.pestis_YopT***  ------------Y----VG--G---R---K---G---KFQIDTLYS------------IKQ--------L-------Q-------------IDG-------C------------KAD--VDQD-EVT-L-DWFKKNGISE

***Y.pseudotuberculosis__Yop*** ------------Y----VG--G---R---K---G---KFQIDTLYS------------IKQ--------L-------Q-------------IDG-------C------------KAD--VDQD-EVT-L-DWFKKNGISE

***Ph.asymbiotica_YopT***  ------------Y----VG--G---Q---K---G---KFHIDTLFS------------IKQ--------L-------Q-------------MDG-------Y------------LD---DEQS-TMT-E-YWLGTQGIQP

***P.temperata_YopT***  ------------Y----VG--G---Q---K---G---QFHIDSLVS------------IKQ--------L-------Q-------------MDG-------I------------AQG--ADQD-TMT-E-SWLSENGIQP

***Ph.luminescens_3_YopT***  ------------Y----VG--G---Q---K---G---KFHIDSLVS------------IKQ--------L-------Q-------------MDS-------Y------------LD---DEQS-TMT-E-YWLGTQGIQP

***Ph.luminescens_2_YopT***  ------------Y----VG--G---Q---K---G---QFHIDSLVS------------IKQ--------L-------Q-------------MDG-------I------------AQD--VDQD-TMT-E-SWLSENGIQP

***Ph.luminescens_1_YopT***  ------------Y----VG--G---Q---K---G---QFHIDSLVS------------IKQ--------L-------Q-------------MDG-------L------------DPY--AEQS-QIT-E-SWLRENGIQP

***Ae.diversa_YopT***  ------------Y----TG--G---Q---K---G---QFQIDTLVS------------IKQ--------L-------Q-------------MDG-------M------------QED--VDQA-RVT-S-NWLKDHNLAP

***V.campbellii_YopT***  ------------F----QS--G---TASDK---G---KLNKETMES------------VAQ--------L-------Q-------------TDG-------M------------VG---YDQE-AIT-E-GWLRSNNIES

***V.harveyi_YopT***  ------------F----QS--G---TASDK---G---KLNKETMQS------------VAQ--------L-------Q-------------TDG-------M------------VG---YDQE-AIT-E-GWLRSNNIES

***Pseudomonas.sp_HopN1***  -----------------NY------R---E---D---ALLP-RVLG------------FQH--------I-------E-------------QQP-------Y------------RDKI-RNAG-PML-L-DTLPKLGMTL

***Ps.avellanae_HopN1***  -----------------DY------R---G---D---ALLP-RVMG------------FQH--------I-------E-------------QQA-------Y------------SNKM-QNAA-PML-L-DTLPKLGMTL

***Ps.syringae_HopN1***  -----------------DY------Q---G---D---ALLP-RVMG------------FQH--------I-------E-------------QQA-------Y------------SNKL-QNAA-PML-L-DTLPKLGMTL

***E.coli_EspL***  -----------------F--------------------------SS------------PQL--------L-------SKAIVIQENKGTNNIKG-------D------------VYFVE-------N-E-SWG-------

***S.flexneri_OspD3***  -----------------F--------------------------AF------------PDE--------I-------KNYVSVSEEEKAINVPA-------I------------IYFVE-------N-G-SWG-------

Core index                                                                                                                                       *

*1581*

*....|....|....|....|....|....|....|....|....|....|....|....|....|....|....|....|....|....|....|....|....|....|....|....|....|....|....|....|*

***E.coli_LifA***  ----------------------------------------------------------------A-WN--QERIT----LL-FN-E----------------------------KG--VKRA-----LI-STPNHTLVLQ

***E.albertii_LifA***  ----------------------------------------------------------------V-WN--PKNLT----QL-FH-E----------------------------KG--VKRA-----LI-TTPTHTLVLQ

***Pr.alcalifaciens_LifA***  ----------------------------------------------------------------S-WQ--PESLV----TL-IL-E----------------------------NG--IQQL-----LV-TTPSHTLVLQ

***C.rodentium_LifA***  ----------------------------------------------------------------S-WQ--PDTLV----TL-FY-K----------------------------KG--IQHL-----LV-TTPTHTLVVQ

***K.pneumoniae_YopT-type***  ----------------------------------------------------------------P-WS--EESLS----IF--F-N----------------------------ND--VNNL-----LI-TTPAHTLIIQ

***Ch.ibidis_LifA***  ----------------------------------------------------------------P-WS--ILTLK----DK-FE-K----------------------------TS--LKSV-----LI-TTPTHSLTLQ

***Ch.buteonis_LifA***  ----------------------------------------------------------------S-WS--LLTLK----DK-FE-K----------------------------TQ--LKSV-----LI-TTPNHSLTLH

***Ch.psittaci_LifA***  ----------------------------------------------------------------P-WS--LLTLK----DK-FE-K----------------------------TQ--LKSV-----LI-TTPNHSLTLH

***Ch.felis_LifA***  ----------------------------------------------------------------N-WD--IPTLS----KF-FETH----------------------------PN--VKSL-----LV-TTPSHSMVLQ

***Ch.suis_LifA***  ----------------------------------------------------------------D-WD--IPNLM----KH-FA-S----------------------------SN--VKSW-----LI-TTPAHSLVLS

***Ch.pecorum_LifA_gene1***  ----------------------------------------------------------------S-WD--IKTLT----HI-FK-T----------------------------EN--TKSL-----LI-TTPTHTLTLS

***Ch.muridarum_LifA_gene1***  ----------------------------------------------------------------K-WD--VLQLT----ST-FE-K----------------------------SL--TKSL-----LI-TTPTHSLTLN

***Ch.pecorum_LifA_gene2***  ----------------------------------------------------------------E-WD--IPSLT----KL-FE-K----------------------------PS--TSSV-----LV-TTPTHVVTVH

***Ch.muridarum_LifA_gene2***  ----------------------------------------------------------------D-WD--IPSLT----KL-FE-K----------------------------SS--VPGV-----LV-TTPSHAVTLH

***Ch.trachomatis_LifA***  ----------------------------------------------------------------E-WD--ITSLI----KL-FE-K----------------------------PV--TPSV-----MV-TTPSHAMTLH

***S.enterica_LifA***  ----------------------------------------------------------------L-DS--PDRVA----QE-IQ-K----------------------------RA--VSAL-----LV-TTGMHSLVIE

***E.coli_ToxB***  ----------------------------------------------------------------L-KD--PVLTE----GI-LY-Q----------------------------RR--ISSL-----LI-TTEYHSLALQ

***G.hollisae_ToxB***  ----------------------------------------------------------------D-FE--TKSVA----DY-LT-D----------------------------TK--VKNL-----LI-TTKFHSMVVS

***E.coli_LifA-like_O111***  ----------------------------------------------------------------D-FN--TASVV----HY-LK-D----------------------------KN--INNI-----LI-TTEHHSFVVS

***E.coli_LifA-like_O127***  ----------------------------------------------------------------D-FN--TASVV----HY-LK-D----------------------------KK--INNI-----LI-TTEYHSFVVS

***Ph.asymbiotica_PaTOX***  ----------------------------------------------------------------N-IN--VSNLATS-ETK-LF-P----------------------------TE--ISSV-----RV-DAPKHTMLIS

***V.vulnificus_McfVv***  V-WQEK----------------------------------LEG-----------------K-Q-A-LT--VNGVVEKIT----D-A-------------------------S-ANG-KPVLL-----EL-DAPGHAMAAW

***Ph.luminescens_Mcf1***  ----QA----------------------------------STE-----------------L-G-Q-FG--LSEVVSRLA----A-T-----------------------------T-GTSMF-----AL-NTQNHSMMVG

***Ph.luminescens_Mcf2***  ----QA----------------------------------STA-----------------R-G-Q-VK--LSEVVSLLK----G-A-----------------------------T-KTSMF-----AL-NTPNHSMMVG

***Azospirillum.sp_peptidase*** -ASS-----------------------R-TEWMS--------A----------------S--D-P-DS--VKEVTKAIATA-TAGT-------------------------H-TYHLVSLHF-----TQ-RGAAHATCCY

***Co.fungivorans_peptidase***  AIYT----------------KTINDLNR-MALHV--------N----------------S--H-SNLR--MEALPS-ALET-I----------------------------S-GYAMLRLSL-----PGRQSPRHTWAIH

***R.solanacearum_peptidase***  S-GKSGMARLDAMAQEQ-----------PSQMLGLTIGGEAH--------------------S-H-KS--VGSTARVLT----E-F-------------------------Q-GY--GLLAARGT-GSR-DGNSHATALH

***R.solanacearum_RipT***  F-GKSDMARLDAIAQEQ-----------PSQILGLTIGTEAY--------------------S-H-KS--VGSTARVLT----E-F-------------------------D-GY--GLLALRMAGSRG-AINGHAAALH

***P.syringae_peptidase***  S-N-A---------------------------RI--------L----------------D--I-M-SD--EEQAMGSMANT-LRDP-------------------------NSSHV--LVIV-----RM-NGDNHAIATH

***Ps.syringae_AvrPphB***  R-REP---------------------------KT--------V----------------H--A-S-GG--SAQLGQTVAHD-VAQS-------------------------GRKHL--LSLR-----FA-NVQGHAIACS

***Ps.syringae_ORF4***  V-GEPS----------------------------------VF--------------------H-A-DR--QSTALQKIA----R-D-------------------------G-SV--HLISLCFE-NNG-KRVRHAITAS

***Ps.savastanoi_ORF4***  V-GEPS----------------------------------VF--------------------H-A-DR--QSTALQKIA----R-D-------------------------G-SA--HLISLCFE-NNG-KRVRHAITAS

***Ac.citrulli_NopT***  A-DNED----------------------------------IY--------------------R-S-DA--LSDVARAVS----T-N-------------------------G-TR--HLLGLY----FT-DGTAHTVATS

***B.japonicum_NopT_1***  A-GEEK----------------------------------RFA-----------------FGK-S-SN--VKSMVNEIN----E-D-------------------------G-SN--HLLSLY----FA-EGGAHTVATS

***Si.fredii_NopT***  S-GKEK----------------------------------VYK-----------------V-G-E-PN--FPRMLTKIT----A-D-------------------------G-SN--HLLSLY----FA-EGGAHTVATS

***B.japonicum_NopT_2***  S-AEET----------------------------------RYR-----------------FGT-S-SC--IDKIVNELA----Q-D-------------------------P-SV--HLVSLKFV--QP-GAGTHTIATA

***Pseudomonas.sp_peptidase***  N-D-------------------------------------F---------------------C-S-EG--LSNAMSMVV---NDDK-------------------------D-GF--NMCYLY----NP-SGPGHAVACI

***Ca.hamiltonella_YopT***  I-DFI----------------------DISKKDD--------E----------------E--R-H-VF--ALRVADSIISN-KNKK-------------------------N-IYE--RIAL-----ET-KNAAHDISIM

***X.bovienii_peptidase***  ----------------------------------------------------------------C-YG--GGSIV----EN-LA-T----------------------------IH--KGYD-----VL-AYGDHAIGLA

***T.oleivorans_peptidase***  H-SSTRI---------PM---------NS--------------------------------------------------------------------GALKTEL---ERIQG-GHV--LIIL---DGQR-ANGSHAISAY

***M.variabilis_peptidase***  E-DRWSS---------SAI--------AV-------------ALNNMVVIQPGQPVPPVQ--PIP-PI--NTSLVNALRS----LR-------------------------D-AYG--YITF-----SG-RRAGHAVAAW

***Sc.paludicola_pepidase***  R-TDMKM---------SK---------VNMSVGG--------K----------------L--Q-K-VN--VANRATASADR-GGGSRMGQGKALGNALISGSMVKNMGLGDG-TYR--MIGI-----HG-SVGGHCMCAF

***Ha.ganghwensis_peptidase***  R-RRITG---------GVPLMGGGHFHHPRVLNG--------Q----------------R--G-R-QS--RSDFSADIAHGITSSS-------------------------G-SYK--MIGI-----EG-SNFAHAMAAW

***Ha.chejuensis_peptidase***  R-KHVSS---------GPSW-------FKGMSRG--------S----------------T--G-D-RGGRRRDFSAEIGRAITSSA-------------------------K-SYK--MIGV-----AG-PNFAHAVAAW

***Hae.ducreyi_LsAp2***  K-QKYFG---------KLS--------RADEVAG--------T----------------V--S-K-ND--VSALVKAILDT-GNES-------------------------S-AVK--KISI-----NL-EGGSHTVSAS

***Hi.somni_IbpA***  K-EKKVA---------DFV--------RRDEVSG--------T----------------V--S-K-ND--VSSLVKAILDT-GDDT-------------------------A-GVK--KISI-----NL-EGGSHTVSAA

***Pa.dagmatis_YopT***  K-EKKVG---------SSS--------RRDEVAG--------T----------------V--S-K-TD--ISALTKAILDT-GSDS-------------------------S-GVK--KISI-----NL-EGGSHTVSAA

***Pa.multocida_peptidase_2***  K-EKKVG---------DLS--------RRDEVAG--------T----------------V--S-K-SD--ISALTKAILDT-GSDT-------------------------A-GAK--KISI-----NL-EGGSHTVSAL

***Pa.multocida_PfhB2***  K-EKKVG---------DLS--------RRDEVAG--------T----------------V--S-K-SD--ISALTKAILDT-GSDT-------------------------A-GAK--KISI-----NL-EGGSHTVSAL

***Pa.multocida_YopT***  K-EKKVG---------STS--------RRDEVAG--------T----------------V--S-K-SD--ISALTKAILDT-GSDI-------------------------S-GVK--KISI-----NL-EGGSHTVSAA

***Pa.multocida***  K-EKKVG---------SAS--------RRDEVAG--------T----------------V--S-K-SD--ISALTKAILDT-GSDI-------------------------S-GVK--KISI-----NL-EGGSHTVSAA

***Y.enterocolitica_YopT***  R-MIERH---------C--------LLRPVDVTG--------T----------------T--E-S-EG--PDQLLNAILDT-HGIG-------------------------Y-GYK--KIYL-----SG-QMSAHAIAAY

***Y.pestis_YopT***  R-MIERH---------C--------LLRPVDVTG--------T----------------T--E-S-EG--LDQLLNAILDT-HGIG-------------------------Y-GYK--KIHL-----SG-QMSAHAIAAY

***Y.pseudotuberculosis__Yop*** R-MIERH---------C--------LLRPVDVTG--------T----------------T--E-S-EG--LDQLLNAILDT-HGIG-------------------------Y-GYK--KIHL-----SG-QMSAHAIAAY

***Ph.asymbiotica_YopT***  N-RQKND---------NM-------NEHSSKIVG--------E----------------T--G-T-RG--TKDLLRAILDT-GDKG-------------------------S-GYK--KISF-----LG-KMAGHTVAAY

***P.temperata_YopT***  R-MKTIT---------YQSANGPIDYKNPIEING--------Q----------------T--G-S-NG--TEDLLNAILDT-GDQG-------------------------S-SYK--KIGF-----SG-QMAGHTVAAY

***Ph.luminescens_3_YopT***  I-MQKND---------V--------DEHSSKVVG--------Q----------------T--G-N-KG--TTDLLRAILDT-GDKG-------------------------S-GYK--KISF-----LG-KMAGHTVAAY

***Ph.luminescens_2_YopT***  R-MKTIT---------YQSANGPINYKRPIEVNG--------Q----------------T--G-S-NG--TSDLLNAILDT-GDKG-------------------------S-SYK--KISF-----SG-QMAGHAVAAY

***Ph.luminescens_1_YopT***  R-SP-------------------------LEVSG--------E----------------T--G-S-KG--TKDLLNAILDT-GDKG-------------------------S-GYK--KISF-----EG-QMAGHTVAAY

***Ae.diversa_YopT***  R-MNKSG---------Y----------PMPPVSG--------Q----------------T--G-A-QG--TDRLVAAILDT-SGEG-------------------------S-EYK--KISF-----SG-KMAAHTVAAH

***V.campbellii_YopT***  P-YRELG---------ALS-----------RVHG--------Q----------------TSRG-R-NG--AAELAAKIVDN-GPKT-------------------------S-LLK--KIGL-----EG-PSNAHAVAAA

***V.harveyi_YopT***  P-YRELG---------ALS-----------RVHG--------Q----------------TSRG-R-NG--AAELAAKIVDN-GPKT-------------------------S-LLK--KIGL-----EG-PSNAHAVAAA

***Pseudomonas.sp_HopN1***  G-K-------------APD--------GVRPAHY--------G----------------M--R-M-DA--IDHELKSVLKP------------------------------G-TNQ----TF-----LL-LSESHAMALH

***Ps.avellanae_HopN1***  G-K-------------GLG--------RAQHAHY--------A----------------V--A-L-EN--LDRDLKALLQP------------------------------G-KDQ----ML-----LF-LSDSHAMALH

***Ps.syringae_HopN1***  G-K-------------GLG--------RAQHAHY--------A----------------V--A-L-EN--LDRDLKAVLQP------------------------------G-KDQ----ML-----LF-LSDSHAMALH

***E.coli_EspL***  -----------------------------------------------------------------------SVIYNLFLQL-EKEN-------------------------K-SH----TSL-----EV-HSPGHAMALG

***S.flexneri_OspD3***  -----------------------------------------------------------------------DIIFYIFNEM-IFHS-------------------------E-KS----RAL-----EI-STSNHNMALG

Core index                      . :*

*1596*

*....|....|....|....|....|....|....|....|....|....|....|....|....|....|....|....|....|....|....|....|....|....|....|....|....|....|....|....|*

***E.coli_LifA***  QL--------E------DIYRLTDPNFGHADFLSPID-----------------------------------------------ALKFI-E--------AMIQ-------------------------------------

***E.albertii_LifA***  QL--------E------DIFRVTDPNFGHADFFSPLD-----------------------------------------------ALKFI-E--------AGIQ-------------------------------------

***Pr.alcalifaciens_LifA***  QL--------E------ERFRVTDPNFGHADFISPWD-----------------------------------------------ALHFI-E--------ASVQ-------------------------------------

***C.rodentium_LifA***  QL--------E------DSFRVTDPNFGHADFASLMD-----------------------------------------------ALHFI-E--------SSVQ-------------------------------------

***K.pneumoniae_YopT-type***  HF--------D------SLYRVTDPNFGHAEFPSLSD-----------------------------------------------AVHFL-E--------AGIQ-------------------------------------

***Ch.ibidis_LifA***  KL-------GE------DIYRLTDPNFGHVDFPAVNQ-----------------------------------------------AFYFV-C--------GIME-------------------------------------

***Ch.buteonis_LifA***  KL-------GA------DVYRLTDPNFGHIDFPSVEK-----------------------------------------------AFYFV-S--------GMME-------------------------------------

***Ch.psittaci_LifA***  KL-------GN------DVYRLTDPNFGHTDFPSVEK-----------------------------------------------AFYFV-S--------GIME-------------------------------------

***Ch.felis_LifA***  PL--------G------STYRVTDPNFGHVDFPSMEA-----------------------------------------------ALYFL-E--------LSVQLSDKI-K--A-R-YGISD-------------D--QPI

***Ch.suis_LifA***  LM--------E------DFFRVTDPNYGHADFPTLEA-----------------------------------------------ALSFL-E--------RMVQ-------------------------------------

***Ch.pecorum_LifA_gene1***  FM--------D------SFFRVTDPNFGHVDFPSIVM-----------------------------------------------ALHFI-E--------EMVQSSDLI-K--M-R-YGISN-------------E--KPI

***Ch.muridarum_LifA_gene1***  FM--------G------SFFRVTDPNFGHVDFPSLAA-----------------------------------------------ALYFI-E--------DMVQ-------------------------------------

***Ch.pecorum_LifA_gene2***  MF--------D------NAVRVTDPNFGHADFPTLEA-----------------------------------------------ALYFI-E--------FMVQVTSEI-K--A-R-YGISD-------------D----K

***Ch.muridarum_LifA_gene2***  FF--------D------GAFRVTDPNFGHVDFPSLES-----------------------------------------------ALYFL-E--------YMVQISSDV-R--A-Q-YGIKE-------------G--VSV

***Ch.trachomatis_LifA***  FW--------D------KTCRVTDPNFGHVDFPSVEA-----------------------------------------------ALHFV-E--------YMVQISEDV-C--K-Q-YGISD-------------D--IPI

***S.enterica_LifA***  QM--------D------TGWRLTDPNFGHGSFATLPE-----------------------------------------------ALTFI-Q--------TV---------------------------------------

***E.coli_ToxB***  QV--------S------SFWRVTDPNFGHCDFHSLAQ-----------------------------------------------ALTFI-K--------NI---------------------------------------

***G.hollisae_ToxB***  AF--------D------GKYRVTDPNFGYADFTSLEQ-----------------------------------------------ALKFV-E--------HSIQ-------------------------------------

***E.coli_LifA-like_O111***  VF--------D------DVVRVTDPNFGYADFSSLEQ-----------------------------------------------SLAFI-E--------NSVS-------------------------------------

***E.coli_LifA-like_O127***  VF--------D------DVVRVTDPNFGYADFSSLEQ-----------------------------------------------SLAFI-E--------NSVS-------------------------------------

***Ph.asymbiotica_PaTOX***  KIKN---RENK------IKYVFYDPNYGMAYFDKHSD-----------------------------------------------MAAFF-Q--------KKMQQYDFP-DDSV-S-FHPLDYSNVSDIKISGRNL--NEI

***V.vulnificus_McfVv***  AKG----SGDD------RVYGFYDPNAGIVEFSSAEK-----------------------------------------------FGDYL-T--------RFFGK-SDL-N--M-A-Q---------------SYK--LGK

***Ph.luminescens_Mcf1***  STV----TTEG------RRYYFYDPNVGIFAFDNTKS-----------------------------------------------LSRAM-E--------QHLVG-RR---------------------------------

***Ph.luminescens_Mcf2***  NTI----GLEG------RRYYFYDPNVGIFAFNDAVS-----------------------------------------------FFRAM-K--------NHLVG-RK---------------------------------

***Azospirillum.sp_peptidase*** KS-------GGKAFGLGSHLYFFDPNYGEFKVSAGSA-----------------------------------------------A-DLF-A--------GLVDRYRHY-E--A-R-DGSTI-------------D--YRV

***Co.fungivorans_peptidase***  NA-------G------QGHMTLFDPNYGEFHVPNHRL-----------------------------------------------P-DFL-K--------TLNEQIKLD----------LGN-------------T--VAQ

***R.solanacearum_peptidase***  RQ------PGS------NHITFFDPNLGEFHIPLHH------------------------------------------------TKDFL-Q--------AYADM-RK--G--L-------------------GQP--VSQ

***R.solanacearum_RipT***  RQ------PGS------SHITFFEPNLGEFHIPLHD------------------------------------------------TKDFL-Q--------AYAGM-QK--S--L-------------------GQP--VSQ

***P.syringae_peptidase***  C--------TG------NKLHVFDPNHGEY--------------------------------------------------------------------------------------------------------------

***Ps.syringae_AvrPphB***  C--------EG------SQFKLFDPNLGEFQSS-----------------------------------------------------------------------------------------------------------

***Ps.syringae_ORF4***  SS--------E------GSVNVFDPNYGEF--------------------------------------------------------------------------------------------------------------

***Ps.savastanoi_ORF4***  SS--------E------GSVNVFDPNYGEFSTTLPE------------------------------------------------LPSMF-Q--------NLMTR-YGS-R--L-------------------NGHLQLES

***Ac.citrulli_NopT***  AA--------G------GKVTLFDPNFGEFEAPPRR------------------------------------------------MGGLM-Q--------SLSNR-YER-P--N-------------------GHI--LMA

***B.japonicum_NopT_1***  AS--------N------GTTTLFDPNYGEFTVRSDPD-----------------------------------------------QMASLLQ--------SLANR-YRN-P--N-------------------GQH--LST

***Si.fredii_NopT***  AM--------D------GNTTLFDPNFGEFTVQSDQ------------------------------------------------IDDLF-R--------SLANR-YSN-P--N-------------------RQH--LTT

***B.japonicum_NopT_2***  TS--------N------GTTILSDPNYGEFTVPSDR------------------------------------------------VGGLF-K--------SLAER-YST-L--N-------------------KRD--ISA

***Pseudomonas.sp_peptidase***  KE--------Q------DCIKFMDPNFGEMSFNSSTE-----------------------------------------------FENWM-S--------NVFDKRYSY-F--S-R-LSVNM-----------YES--PSN

***Ca.hamiltonella_YopT***  IEN----QKNK------TKVIFSDPNHRLFTFYHEED-----------------------------------------------FKAWF-I--------YFCLNH--YW-------------------------------

***X.bovienii_peptidase***  VT--------A------NGIDFFDPNFGCVHFPTGHN-----------------------------------------------FRTWF-K--------EKYWPNRDG--IGA-I-ARPCDKIIVHSIA-----------

***T.oleivorans_peptidase***  IG-------QN-----GEDVCFFDPNYGEYWFQNR---------------------------------------------------------------------------------------------------------

***M.variabilis_peptidase***  VA-------DDRV-NSDIGALFFDPNYGEYRFATKGD-----------------------------------------------FLNFF-D--------AYYR-HAYM-R--G-W-IQFRS-------------D--WSV

***Sc.paludicola_pepidase***  V---------------GQDVCFFDPNFGEFYFPKRDD-----------------------------------------------FAKWF-G--------DYFWPKSFY-D--W-AL------------------------

***Ha.ganghwensis_peptidase***  -S--------------DQDVSFFDPNFGEFWFARPED-----------------------------------------------FHAWF-P--------RFWQ-LAGY-A--A-PM------------------------

***Ha.chejuensis_peptidase***  -S--------------DTDVTFFDPNFGEFWFPTTTA-----------------------------------------------FQGWF-P--------RFWH-LAGY-G--T-PAIGLSE-------------S--YEI

***Hae.ducreyi_LsAp2***  IE-------G-------QKVVFFDPNFGEITFKDKKS-----------------------------------------------FEKWM-K--------NAFWKKSGY-A--G-K-KDTKR-------------F--FNV

***Hi.somni_IbpA***  VD-------G-------SKVTFFDPNFGEMTFPTHQQ-----------------------------------------------FENWL-K--------NAFWQKSGY--------------------------------

***Pa.dagmatis_YopT***  IQ-------G-------QKVVFFDPNFGEMTFPSHKQ-----------------------------------------------FESWL-K--------GAFWEKSG---------------------------------

***Pa.multocida_peptidase_2***  VQ-------G-------EKVVFFDPNFGEMTFPSHQK-----------------------------------------------FESWL-K--------EAFWEKSGY--------------------------------

***Pa.multocida_PfhB2***  VQ-------G-------EKVVFFDPNFGEMTFPSHQK-----------------------------------------------FESWL-K--------EAFWEKSGY-A--G-K-KEGKR-------------F--FNV

***Pa.multocida_YopT***  VQ-------G-------QKVVFFDPNFGEMTFPSHQK-----------------------------------------------F-------------------------------------------------------

***Pa.multocida***  VQ-------G-------QKVVFFDPNFGEMTFPSHQK-----------------------------------------------FETWL-K--------EAFWNKSG---------------------------------

***Y.enterocolitica_YopT***  VN-------EK------SGVTFFDPNFGEFHFSDKEK-----------------------------------------------FRKWF-T--------NSFWENSMY-H--Y-P-LGVGQ-------------R--FSV

***Y.pestis_YopT***  VN-------EK------SGVTFFDPNFGEFHFSDKEK-----------------------------------------------FRKWF-T--------NSFWGNSMY-H--Y-P-LGVGQ-------------R--FRV

***Y.pseudotuberculosis__Yop*** VN-------EK------SGVTFFDPNFGEFHFSDKEK-----------------------------------------------FRKWF-T--------NSFWDNSMY-H--Y-P-LGVGQ-------------R--FRV

***Ph.asymbiotica_YopT***  VD-------DQ------KGVTFFDPNFGEFNFPDKVS-----------------------------------------------FSHWF-T--------DDFWPKSWY-S--L-E-IGLGQ-------------E--FEV

***P.temperata_YopT***  VD-------DQ------KGVTFFDPNFGEFNFPDKTS-----------------------------------------------FSNWF-T--------QDFWPKSMY-N--M-E-IGLGQ-------------H--FQV

***Ph.luminescens_3_YopT***  VD-------DQ------KGVIFFDPNFGEFSFPSITS-----------------------------------------------FSRWF-T--------DDFWPKSWY-N--L-E-IGLGQ-------------Q--FEV

***Ph.luminescens_2_YopT***  VD-------DQ------KGVTFFDPNFGEFHFPNKES-----------------------------------------------FSNWF-S--------QDFWSKSMY-N--K-E-IGLGQ-------------H--FQV

***Ph.luminescens_1_YopT***  VD-------DQ------KGVTFFDPNFGEFSFPDKTS-----------------------------------------------FSNWF-S--------QDFWSKSMY-N--K-E-IGLGQ-------------N--FYV

***Ae.diversa_YopT***  VD-------EQ------QGVTFFDPNFGEFHFPDKEQ-----------------------------------------------FKNWF-T--------DAFWPGSKY-K--L-E-IGLGQ-------------E--FEV

***V.campbellii_YopT***  VD-------G-------EKVTFFDPNFGEFSFPSKED-----------------------------------------------FTSWF-V--------QDFWHKSGY-D--LPK-FGLSG-------------E--FSI

***V.harveyi_YopT***  VD-------G-------EKVTFFDPNFGEFSFPSKED-----------------------------------------------FTSWF-V--------QDFWHKSGY-D--LPK-FGLSG-------------E--FSI

***Pseudomonas.sp_HopN1***  QD-------SQ------NRLHFFDPLFGVVQSDNREN-----------------------------------------------MSSFL-C--------DVF--------------------------------------

***Ps.avellanae_HopN1***  QD-------SQ------GCLHFFDPLFGVVQADSFSN-----------------------------------------------MSHFL-A--------DVF--------------------------------------

***Ps.syringae_HopN1***  QD-------SQ------GCLHFFDPLFGVVQADSFSN-----------------------------------------------MSHFL-A--------DVF--------------------------------------

***E.coli_EspL***  IKIKND-KENK------FVINFYDPNQTATHKRVFFCTNNICDIINLTAYDFLSEQCLKCYGLKEDTLSLFVDKTKSNDNNNVFIKKLP-D--------NILQGVVIN-F-AM-G-AGLRE-------------I--IKK

***S.flexneri_OspD3***  LKIKETKNGGD------FVIQLYDPNHTATHLRAEFNKFNLAKIKKLTVDNFLDEKHQKCYGLISDGMSIFVDRHTPT------------SMSSIIRWPNNLLHPKVIYH-AM-R-MGLTE-------------L—IQK

**Figure S1 The C58 cysteine protease domain (CPD) is conserved in lymphostatin.** Partial sequence alignments of MEROPS class C58 CPDs, LifA and LifA-like CPDs assembled from PSI-BLAST [1] searches against the CPD of LifA (W1442 – Q1619). Sequences were aligned with Psi-Coffee [3]. The sequences are annotated with a bar above the sequences: **BAD AVG GOOD** to show high local residue consistency (core index). Amino acids close to the catalytic triad of each CPD (LifA: C1480, H1581 and D1596) are best aligned (red text). The best aligned residues represent the core of the papain-like fold and define the active site (see Figure S2). Coloured bars, to the left of the name, relate to the phylogenetic tree illustrated in Figure 2 (main paper). Q1470, C1480, H1581 and D1596 from LifA are indicated with black arrows. * mark the catalytic residues in lymphostatin. Database acession numbers, full species names and sequence positions of the catalytic residues are shown in Table S1.


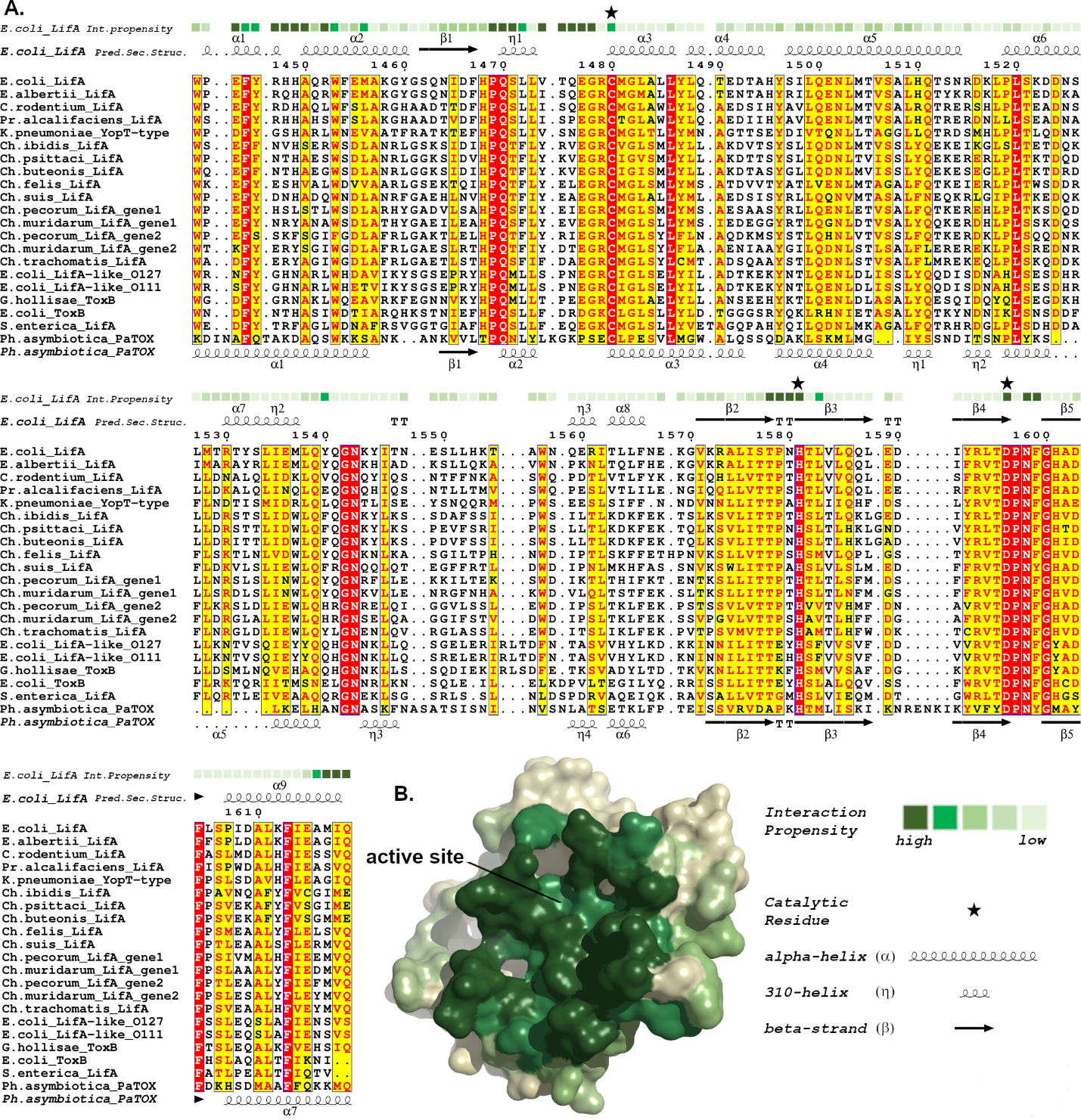


**Figure S2. Sequence alignments show LifA-like proteins represent a well defined subset of C58 cysteine protease domains (CPDs).** (a) Partial sequence alignments of the CPD of LifA-like proteins from pathogenic bacteria assembled in T-Coffee Expresso [13]. The CPD of PaTox was used as the structural template (PDB ID: **6HV6**). The predicted secondary structure of the LifA CPD is shown above the sequence [4]. The secondary structure of PaTox, derived from crystallographic data, is shown at the bottom of the sequences. Sequence numbers refer to LifA. Catalytic residues are annotated with an asterisk. The bar at the top of the sequences represents the residue interaction propensity calculated in the docking program ArDock [12]. (b) ArDock interaction propensity mapped to our homology model of the LifA CPD. The figure was created in ESPript 3.0 [14]. Sequence identity is indicated by a red background, white text and similarity with red text and a yellow background.


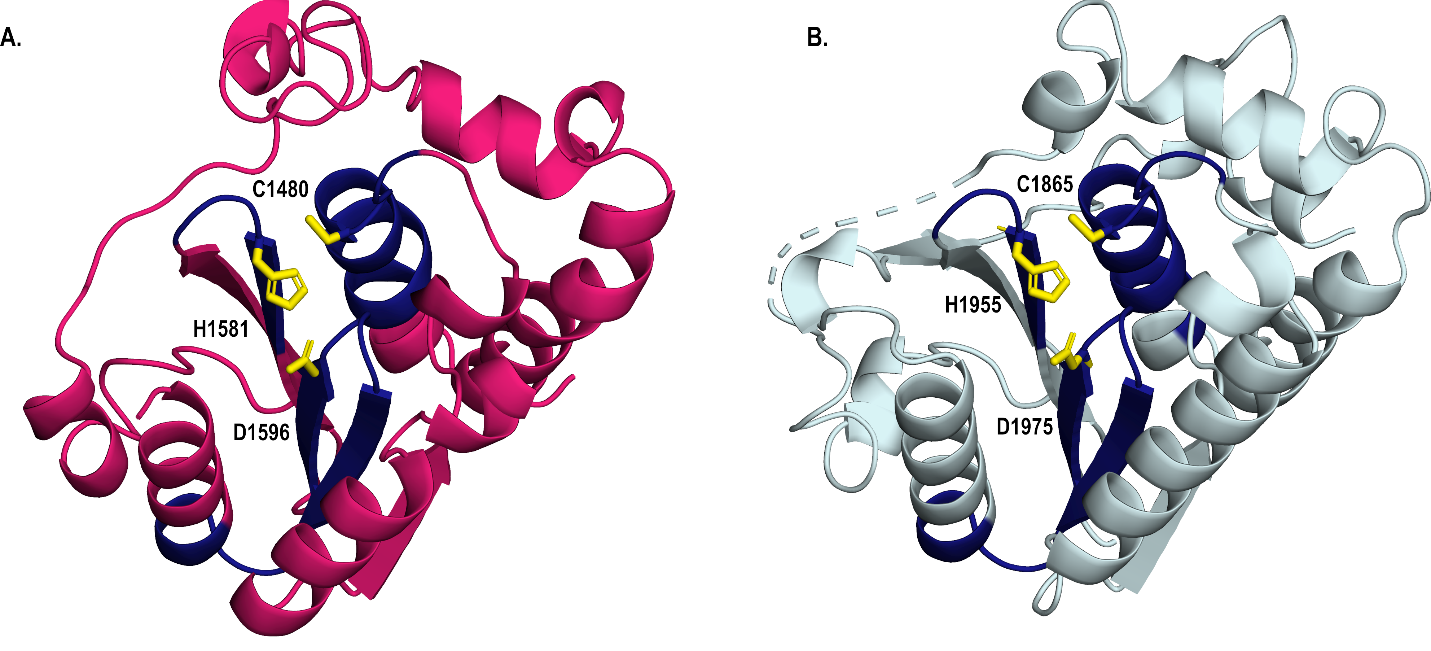


**Figure S3. Residues with the highest sequence conservation define the putative active site of the lymphostatin CPD.** (a) A homology model of the C58 CPD of LifA (W1442 – Q1619) based on PaTox (PDB ID: **6HV6**) was constructed using the Phyre2 server [11]. The residues with highest sequence conservation score in Figure S1 are coloured dark blue. Catalytic residues are shown as yellow sticks*.* (b) The CPD of PaTox is similarly annotated (K1825 – D2032). Figures were prepared in PyMol (The PyMOL Molecular Graphics System, Version 2.4.0 Schrödinger).

**
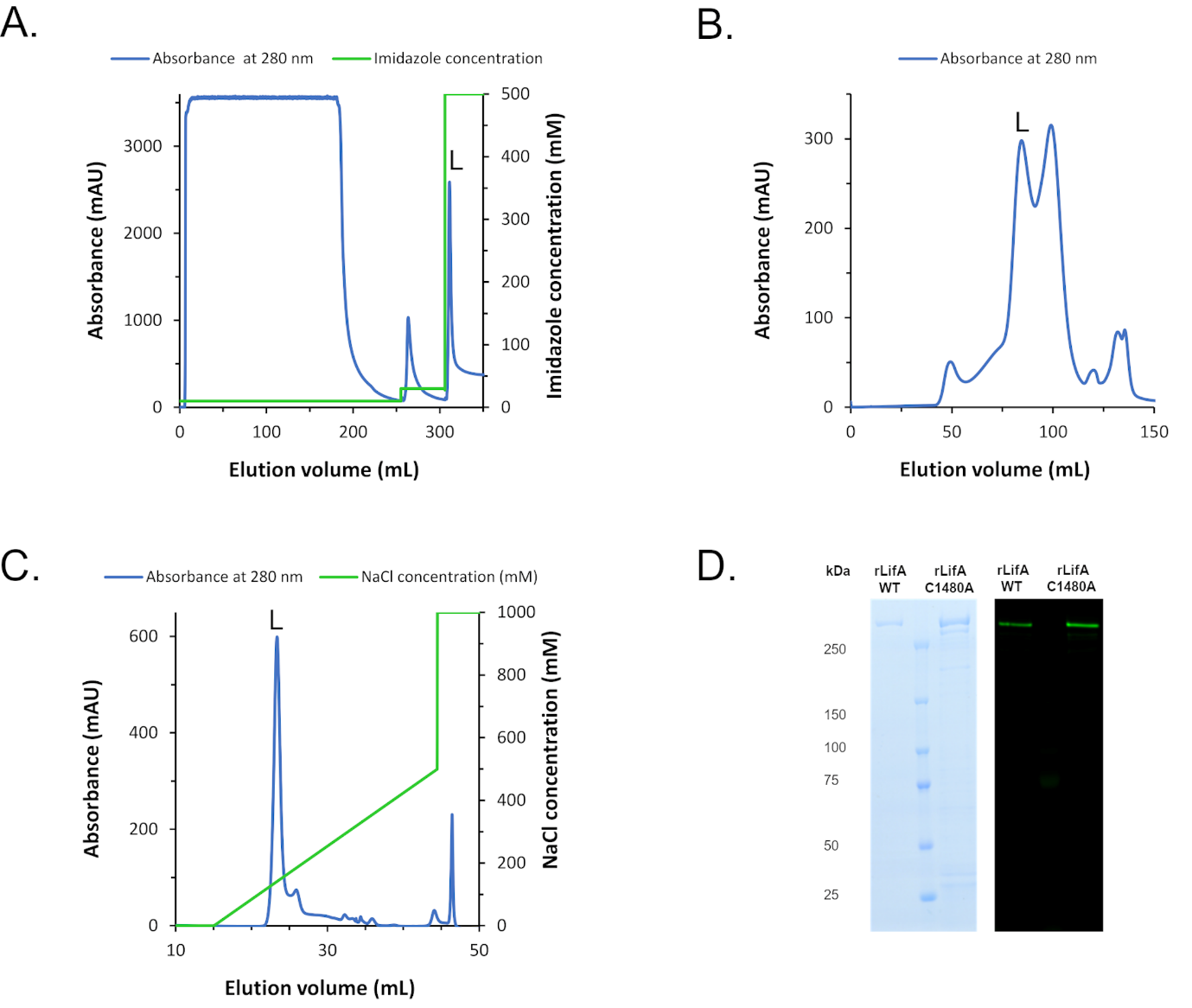
**

**Figure S4. Multiple-step purification of rLifA^C1480A^ yields a highly pure full-length protein.** (a) Ion metal affinity chromatography (HiTrap IMAC FF, GE Healthcare) elution profile of rLifA^C1480A^; lymphostatin (L) was eluted over a 20 – 500 mM imidazole step after extensive washing. (b) Size-exclusion chromatography (Superose-6pg XK 16/60, GE Healthcare) separates rLifA^C1480A^ from lower molecular mass contaminants. (c) Anion exchange chromatography (Mono-Q 5/50 GL, GE Healthcare) was used to remove remaining contaminants; rLifA^C1480A^ was eluted at ~140 mM NaCl. (d) Coomasie stain (left) and western blot using rabbit polyclonal anti-LifA antibody (right) of anion exchange-purified WT rLifA and rLifA^C1480A^.


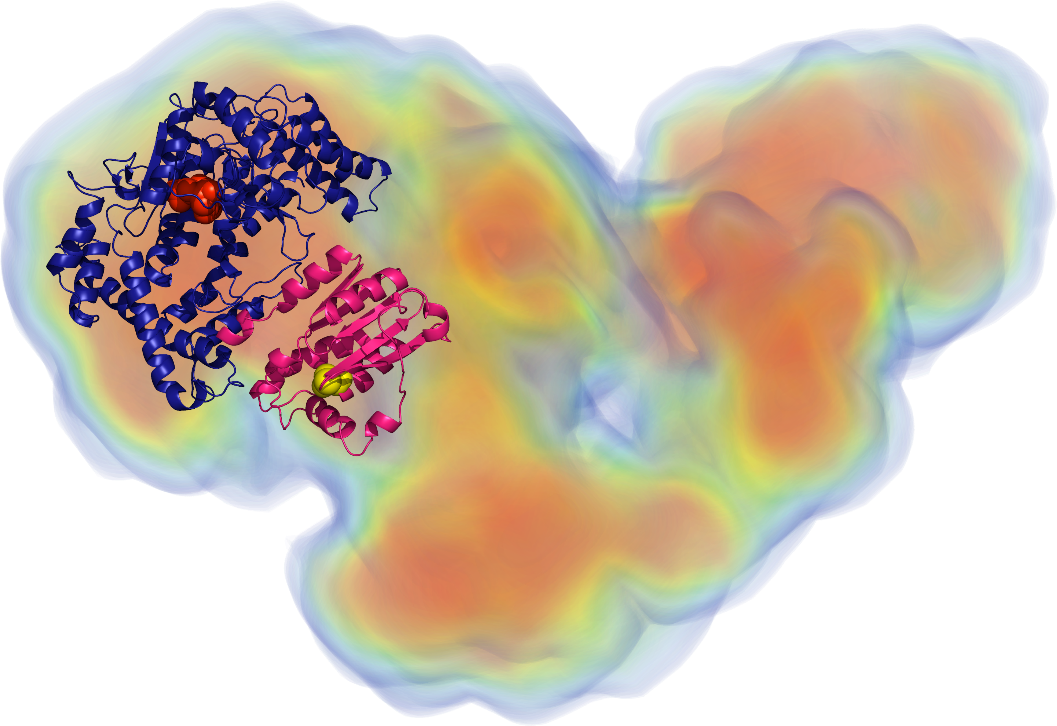


**Figure S5. Comparing homology models of the glycosyltransferase and cysteine protease domains of lymphostatin to 3D electron density maps calculated from solution scattering.** Homology models of the glycosyltransferase domain (A242 – R769) shown in blue cartoon representation (described in [2]) and the putative C58 CPD of lymphostatin (W1442 – Q1619) shown in green cartoon orientated by alignment to the crystal structure of TcdA from Clostridium difficile (PDB ID: **4R04**) were fitted to the electron density map generated in DENSS using the Fit in Map tool in the program UCSF chimera [15,16]. The electron density map is shown as a volume and coloured according to density; red>orange>yellow>green>blue. The atoms of the DTD glycosyltransferase motif are highlighted as red spheres and C1480 is highlighted as yellow spheres. This domain arrangement both fits well in terms of putative electron density and exposes the active sites of the glycosyltransferase and cysteine protease domains to solvent where they are accessible to substrate. Figures were prepared in PyMol (The PyMOL Molecular Graphics System, Version 2.4.0 Schrödinger, LLC).

**Table S2. Size-exclusion elution volumes and geometric parameters derived from small angle X-ray scattering (SAXS) for rLifA and rLifA^C1480A^ as a function of pH.** WT rLifA and rLifA^C1480A^ elute later in size-exclusion chromatography under acidic conditions. This is consistent with a change in stokes radius/hydrodynamic character. SAXS confirms that both WT rLifA and rLifA^C1480A^ are monomeric in solution at pH 5.5 and pH 7.5. The change from neutral to acidic pH does not induce any gross structural changes but results in a small but measurable increase in flexibility under acidic conditions. R_g_, radius of gyration derived from the Guinier plot and P(r) distribution; MW^seq^, molecular weight calculated from sequence and MW^SAXS^ estimated from scattering; D_max_, maximum dimension of the protein; V_p_, protein volume derived from the Porod analysis; Porod^exp^, the Porod exponent, a measure of particle flexibility. SAXS measurements were carried out on beamline B21 at Diamond Light Source, Didcot, UK; Experiment. No. SM18931-1; fixed camera length configuration (4.014 meters); 12.4 keV beam. A volume of 45 µL of 2 mg/mL WT rLifA and rLifA^C1480A^ mutant protein were loaded onto a Superose 6 Increase 3.2/300 size-exclusion chromatography column (GE Healthcare) pre-equilibrated in 50 mM Bis-Tris, pH 5.5 or 7.5, 100 mM NaCl, 3 % (v/v) glycerol; 15 °C at a flow rate of 0.075 mL/min using an Agilent 1200 HPLC system. Small angle X-ray scattering was collected on the sample as it eluted from the column in 10 s acquisition blocks. SAS data reduction was performed in the DLS software pipeline; ScÅtter (v3.1v) [17]. Analysis of SEC-SAXS data segments was carried out in ScÅtter (http://www.bioisis.net/scatter). Simple geometric parameters were calculated using ScÅtter. Data has been deposited in the Small Angle Scattering Biological Data Bank (SASDB) https://www.sasbdb.org/ [18].

| Protein | pH | elut. (min) | R_g_ (Å) Guinier | R_g_ (Å) P(r) | D_max_ (Å) | V_p_ (Å^3^) | MW^seq^ | MW^SAXS^ | Porod ^exp.^ | SASBDB |
| --- | --- | --- | --- | --- | --- | --- | --- | --- | --- | --- |
| **rLifA** | 7.5 | 21.43 | 58.7 ± 1.2 | 59.4 ± 2.0 | 193 | 559196 | 367 | 398 | 3.75 | **SASDKL9** |
| **rLifA^C1480A^** | 7.5 | 21.31 | 58.9 ± 1.7 | 59.8 ± 0.0 | 191 | 557285 | 367 | 391 | 3.66 | **SASDKN9** |
| **rLifA** | 5.5 | 22.23 | 58.4 ± 1.7 | 58.8 ± 0.0 | 197 | 564540 | 367 | 396 | 3.57 | **SASDKM9** |
| **rLifA^C1480A^** | 5.5 | 22.14 | 58.4 ± 1.0 | 58.9 ± 1.1 | 193 | 560263 | 367 | 404 | 3.54 | **SASDKP9** |


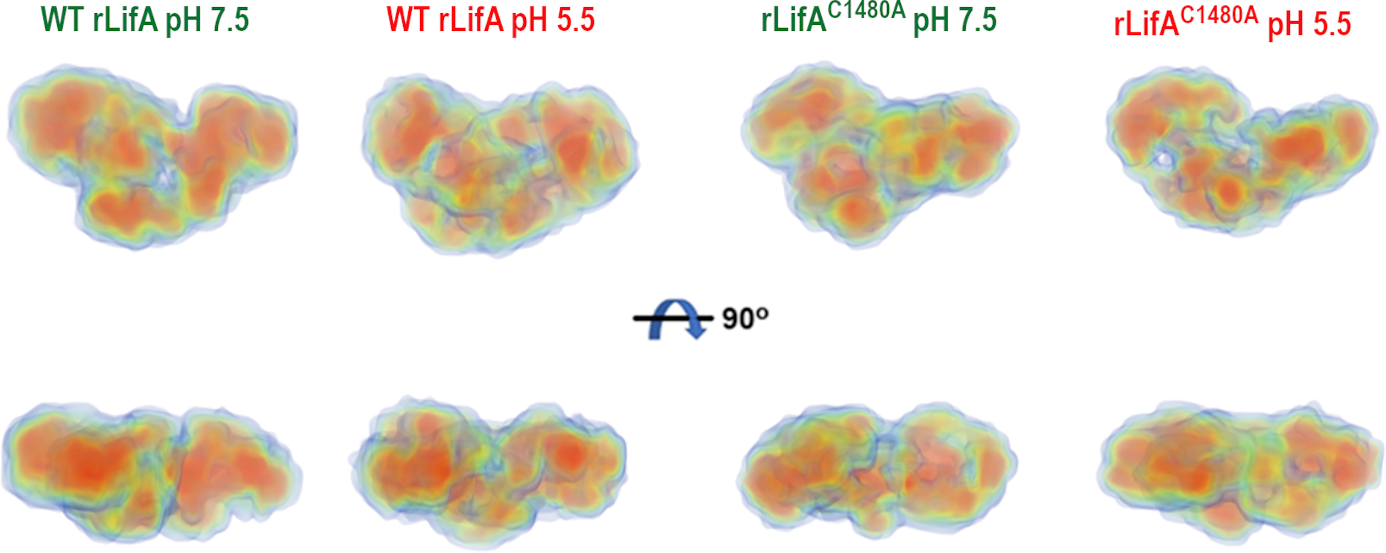


**Figure S6. Electron density maps calculated from solution scattering for WT rLifA and rLifA^C1480A^ proteins at pH 7.5 and pH 5.5**. Electron density maps generated from solution scattering using DENSS [13]. Alignment was performed in DENSS. Electron density maps are shown as volumes and coloured according to density; red>orange>yellow>green>blue. Figures were prepared in PyMol (The PyMOL Molecular Graphics System, Version 2.4.0 Schrödinger, LLC)

**Figure S7. Lymphostatin is processed by J774A.1 cells into N-terminal c. 140 kDa and C-terminal c. 225 kDa species.** Western blots of lysates from J774A.1 macrophage-like cells (1.2 x 10^7^ cells) treated with WT rLifA and rLifA^C1480A^ for 1 h. A volume equivalent to 2.5 x 10^5^ cells was run in each well. (a) Anti-LifA western blot. The c. 140 kDa fragment previously observed in T cells as well as the predicted c. 225 kDa fragment are marked with arrows. (b) The c. 225 kDa fragment is reactive to anti-6 x His antibody (marked by arrows).

**
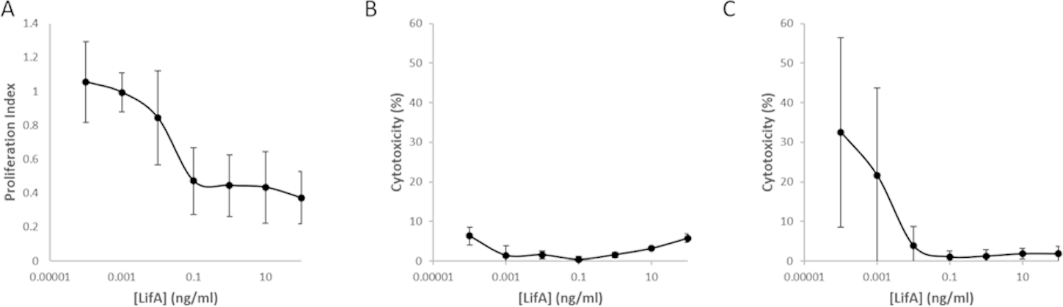
**

**Figure S8. LifA has no cytotoxic activity at the concentration ranges at which it inhibits cellular proliferation.** In parallel with demonstrating the ability of LifA to inhibit cellular proliferation (a), its cellular cytotoxicity was determined at 24 hours (b) and 72 hours (b) of incubation. Samples were tested in triplicate for each condition per donor and the results are the mean obtained from 3 independent experiments using separate donors. Error bars indicate the standard deviation.

**Table S2. ED_50_ values for individual lymphocyte donors in the proliferation assay.** These data are associated with the proliferation data presented in Figure 8 in the main body of the paper. Individual, average and standard error for five bovine calf donors. A one sample T-test with the difference between the rLifA and rLifAC1480A ED_50_ values, transformed by log10, using a hypothesised mean of 0 gave a P value of 0.003.

|  | ED_50_ (ng/mL) | |
| --- | --- | --- |
| **Donor** | **rLifA** | **rLifA^C1480A^** |
| 603349 | 0.0106990 | 25.205218 |
| 103465 | 0.0109422 | 3425.01942 |
| 703471 | 0.0290718 | 140.07877 |
| 103451 | 0.0078291 | 2218.6569 |
| 203459 | 0.0102343 | 267.480909 |
|  |  |  |
| Average | 0.0137553 | 1215.2882434 |
| STER | 0.00386889 | 684.1188601 |

**References**

1. Altschul, S. F., Madden, T. L., Schäffer, A. A., Zhang, J., Zhang, Z., Miller, W. *et al*. (1997). Gapped BLAST and PSI-BLAST: a new generation of protein database search programs. *Nucleic Acids Res.* **25**, 3389-3402 <https://doi.org/10.1093/nar/25.17.3389>
2. Cassady-Cain, R. L., Blackburn, E. A., Alsarraf, H., Dedic, E., Bease, A. G., Böttcher, B. *et al*. (2016). Biophysical characterization and activity of lymphostatin, a multifunctional virulence factor of attaching and effacing *Escherichia coli*. *J. Biol. Chem.* **291**, 5803-5816 <https://doi.org/10.1074/jbc.M115.709600>
3. Notredame, C., Higgins, D. G. and Heringa, J. (2000). T-Coffee: A novel method for fast and accurate multiple sequence alignment. *J. Mol. Biol.* **302**, 205-217 <https://doi.org/10.1006/jmbi.2000.4042>
4. Jones, D. T. (1999). Protein secondary structure prediction based on position-specific scoring matrices. *J. Mol. Biol.* **292**, 195-202 <https://doi.org/10.1006/jmbi.1999.3091>
5. R Core Team (2017). R: A language and environment for statistical computing. R Foundation for Statistical Computing, Vienna, Austria. URL [https://www.R-project.org/](https://www.r-project.org/).
6. Paradis, E. and Schliep, K. (2019). “ape 5.0: an environment for modern phylogenetics and evolutionary analyses in R.” Bioinformatics, **35**, 526-528. <https://doi.org/10.1093/bioinformatics/bty633>
7. Charif, D. and Lobry, J. (2007). “SeqinR 1.0-2: a contributed package to the R project for statistical computing devoted to biological sequences retrieval and analysis.” In Bastolla, U., Porto, M., Roman, H. and Vendruscolo, M. (eds.), Structural approaches to sequence evolution: Molecules, networks, populations, series Biological and Medical Physics, Biomedical Engineering, 207-232. Springer Verlag, New York. ISBN : 978-3-540-35305-8.
8. Bodenhofer, U., Bonatesta, E., Horejs-Kainrath, C. and Hochreiter, S. (2015). “msa: an R package for multiple sequence alignment.” Bioinformatics **31**, 3997–3999. doi: [10.1093/bioinformatics/btv494](https://doi.org/10.1093/bioinformatics/btv494).
9. Letunic, I. and Bork, P. (2019). Interactive Tree Of Life (iTOL) v4: recent updates and new developments. *Nucleic Acids Res.* **47**, W256-W259 <https://doi.org/10.1093/nar/gkz239>
10. McQuade, R. and Stock, S. P. (2018). Secretion systems and secreted proteins in Gram-negative entomopathogenic bacteria: their roles in insect virulence and beyond. *Insects.* **9**, E68 <https://doi.org/10.3390/insects9020068>
11. Kelly, L. A., Mezulis, S., Yates, C. M., Wass, M. N. and Sternberg, M. J. (2015). The Phyre2 web portal for protein modeling, prediction and analysis. *Nat. Protoc.* **10**, 845-858 <https://doi.org/10.1038/nprot.2015.053>
12. Reille, S., Garnier, M., Robert, X., Gouet, P., Martin, J. and Launay, G. (2018). Identification and visualization of protein binding regions with the ArDock server. *Nucleic Acid Res.* **46**, W417-W422 <https://doi.org/10.1093/nar/gky472>
13. Armougom, F., Moretti, S., Poirot, O., Audic, S., Dumas, P., Schaeli, B. *et al*. (2006). Expresso: automatic incorporation of structural information in multiple sequence alignments using 3D-Coffee. *Nucleic Acids Res.* **34**, W604-W608 <https://doi.org/10.1093/nar/gkl092>
14. Robert, X. and Gouet, P. (2014). Deciphering key features in protein structures with the new ENDscript server. *Nucleic Acids Res.* **42**, W320-W324 <https://doi.org/10.1093/nar/gku316>
15. Pettersen, E. F., Goddard, T. D., Huang, C. C., Couch, G. S., Greenblatt, D. M., Meng, E. C. *et al*. (2004). UCSF chimera – A visualization system for exploratory research and analysis. *J. Comp. Chem*. **25**, 1605-1612 <https://doi.org/10.1002/jcc.20084>
16. Grant, T. D. (2018). *Ab initio* electron density determination directly from solution scattering data. *Nat. Methods.* **15**, 191-193 [https://doi.org/10.1038/nmeth.4581](https://www.nature.com/articles/nmeth.4581)
17. Rambo, R. P. and Tainer, J. A. (2013). Accurate assessment of mass, models and resolution by small-angle scattering. *Nature.* **496**, 477-481 <https://doi.org/10.1038/nature12070>
18. Valentini, E. Kikhney, A.G., Previtali, G. Jeffries, C.M. Svergun, D.I. (2015). SASBDB, a repository for biological small-angle scattering data. *Nucleic Acids Res.* **43**, D357-D363 <https://doi.org/10.1093/nar/gku1047>
